# Science of the Van Allen Probes Science Operations Centers

**DOI:** 10.1007/s11214-022-00919-x

**Published:** 2022-11-16

**Authors:** Jerry W. Manweiler, Aaron Breneman, Jonathan Niehof, Brian Larsen, Giuseppe Romeo, Grant Stephens, Alexa Halford, Craig Kletzing, Lawrence E. Brown, Harlan Spence, Geoff Reeves, Reiner Friedel, Sonya Smith, Ruth Skoug, Bern Blake, Dan Baker, Shri Kanekal, Vaughn Hoxie, Allison Jaynes, John Wygant, John Bonnell, Danielle Crawford, Matina Gkioulidou, Louis J. Lanzerotti, Donald G. Mitchell, Andrew Gerrard, Aleksandr Ukhorskiy, Thomas Sotirelis, Robin J. Barnes, Robyn Millan, Blaine Harris

**Affiliations:** 1 RBSPICE, Fundamental Technologies, LLC, Lawrence, KS USA; 2 EFW, University of Minnesota, Minneapolis, MN USA; 3 ECT, University of New Hampshire, Durham, NH USA; 4 ECT, Los Alamos National Laboratory, Los Alamos, NM USA; 5 RBSPICE and Project, Applied Physics Laboratory, The Johns Hopkins University, Laurel, MD USA; 6 BARREL, Dartmouth College, Hanover, NH USA; 7 EMFISIS, University of Iowa, Iowa City, IA USA; 8 ECT, Aerospace Corporation, Los Angeles, CA USA; 9 ECT, Laboratory for Atmospheric and Space Physics, University of Colorado, Boulder, CO USA; 10 ECT, Goddard Spaceflight Center, Greenbelt, MD USA; 11 EFW, University of California-Berkley, Berkley, CA USA; 12 RBSPICE, New Jersey Institute of Technology, Newark, NJ USA

**Keywords:** Magnetosphere, Ring current, Van Allen probes, NASA, Space physics, ECT, EFW, EMFISIS, RBPICE, Van Allen probes gateway, Data analysis, Science operations centers

## Abstract

The Van Allen Probes mission operations materialized through a distributed model in which operational responsibility was divided between the Mission Operations Center (MOC) and separate instrument specific SOCs. The sole MOC handled all aspects of telemetering and receiving tasks as well as certain scientifically relevant ancillary tasks. Each instrument science team developed individual instrument specific SOCs proficient in unique capabilities in support of science data acquisition, data processing, instrument performance, and tools for the instrument team scientists. In parallel activities, project scientists took on the task of providing a significant modeling tool base usable by the instrument science teams and the larger scientific community. With a mission as complex as Van Allen Probes, scientific inquiry occurred due to constant and significant collaboration between the SOCs and in concert with the project science team. Planned cross-instrument coordinated observations resulted in critical discoveries during the seven-year mission. Instrument cross-calibration activities elucidated a more seamless set of data products. Specific topics include post-launch changes and enhancements to the SOCs, discussion of coordination activities between the SOCs, SOC specific analysis software, modeling software provided by the Van Allen Probes project, and a section on lessons learned. One of the most significant lessons learned was the importance of the original decision to implement individual team SOCs providing timely and well-documented instrument data for the NASA Van Allen Probes Mission scientists and the larger magnetospheric and radiation belt scientific community.

## Introduction

The Van Allen Probes Mission Science Operation Centers (SOC) were designed to provide the highest level of science return in one of most intense radiation environments to fly an operational mission around Earth. The success of the Van Allen Probes Mission (Fox and Burch 2014) is reflected by the simple fact that this mission has become the Gold Standard for NASA missions in regards to mission operations successes and scientific return. The sheer number of new discoveries accomplished by the research teams are too numerous to fully describe but a simple metric of this statement can easily be gleaned by doing a search on scientific papers published using the data from Van Allen Probes instruments; the details of the metrics of the scientific success of the Van Allen Probes Mission can be found in the introduction by Ukhorskiy et al. (in prep). Science operations for this mission were broken into multiple levels that included command and control of the spacecraft and instruments; receipt of telemetry; processing of telemetry into higher level data products. The Mission Operations Center (MOC) as described in Kirby et al. (2013), managed communications between the ground segment and each spacecraft; handled spacecraft operations; and provided detailed ephemerid of the spacecraft for each of the instrument teams. The overall configuration of the Van Allen Probes operations was architected using a “bent-pipe” system where the MOC handled all elements related to the spacecraft and the instrument Science Operation Centers (SOC) handled all aspect of instrument operations.

The success of the extension of the standard satellite “bent-pipe” architecture for the data systems coupled with the distribution of operational responsibilities between the central MOC and the instrument SOC’s cannot be overstated. This configuration provided the highest level of flexibility for the instrument teams especially in situations where rapidly changing spacecraft and instrument conditions required very fast response in order to capture the highest telemetry rates and best quality of data or in some situations in order to protect the health of the instrument. As telemetry was processed into higher level data products, each team provided an instrument scientist and/or a data scientist to verify and validate the resulting data products. Since this responsibility was given to each instrument team and not a centralized production center, the scientists involved in the production of each specific data product had a clearer understanding of the specifics of the instrumentation than might have been in other situations and the production software could be quickly modified to handle changing flight configurations as opposed to the teams submitting change order requests to a centralized production center to be implemented, tested, and verified.

With all of this in mind, this paper provides and documents necessary updates for each of the Instrument Science Operations Centers (SOCs) as to any changes, development of software, and operations at the end of the mission. The following sections describe instrument configuration changes and other details for the following topical categories:

1. Post Launch Instrument and SOC Modifications
2. Science Coordination activities
3. Science Analysis Software
4. Science Gateway
5. Lessons Learned

It should be noted that in some instances the complexity of instrument operations and SOC operations was difficult to separate between the instrument papers of this journal and this paper. If the reader cannot find the desired information for which they are searching about a particular instrument, then they should also review the details provided in each of the specific instrument chapters of this journal.

## Post Launch Instruments and SOC Modifications

The Van Allen Probes Mission started mission development with the announcement in May of 2006 of the selection of the Johns Hopkins Applied Physics Laboratory to build and operate the twin Radiation Belt Storm Probes spacecraft. The instrument selections were subsequently announced in the early July of 2006. Preliminary mission design, Mission Phase B, occurred from instrument selection through 2008. The formal Mission Phases C and D occurred from Jan 2009 to launch. During phase C/D, software design and development efforts were underway with the desire to support launch sometime in 2012. The software design and development required the instrument teams to build as flexible a software system as possible for the instrument specific targeted requirements. With the launch of the spacecraft in August of 2012, the instrument teams and SOC teams were required to shift into operations support no matter what the condition of the software systems. In many cases the primary software production systems for the higher- level data products were still in development and in some cases still in design. Delay in development of the higher-level data products occurred in some instances because the teams needed to understand the instrument performance and have a reasonable understanding of the scientific capabilities before attempting to fully specify higher level data products.

In this light it becomes understandable that changes were necessary to both instrument operations and SOC software to accommodate and adapt to the flight of the instruments in what is considered one of the most hostile environments for spacecraft operations in the solar system. The following subsections attempt to describe changes to the SOC’s operationally and/or software configuration post launch as instrument performance and the radiation belt environments were better understood.

One specific project wide decision that was made toward the end of the Critical Design Review stage of the instruments SOC development was the need for a production format that was easily utilized by the instrument teams and the outside scientists whom would be using the various data products. The instrument teams selected the Common Data Format (CDF, see GSFC CDF Main Website). The criteria used by the teams include the following positive reasons for the selection:

1. CDF is a self-documenting data format containing data and metadata,
2. CDF stores data in a platform independent format (using the CDF software libraries),
3. CDF is widely used in the Heliophysics domain,
4. CDF is capable of easily handling multidimensional data (max is 10 dimensions),
5. CDF files can be compressed at the file level or variable by variable,
6. CDF data is accessible via the CDF Software Suite which is distributed in many different programming languages and integrated into many different Commercial Software Programs such as L3HARRIS^™^ Corporations Interactive Data Language or IDL^©^ (IDL Main Website), MathWorks^®^ analysis software MATLAB^©^ (MATLAB Main Website), Wolfram Research software Mathematica^©^ (Mathematica Main Website), Faden et al. (2010) (Autoplot Main Website), and others include customized software programmed by the Van Allen Probes instrument teams for purposes of production and analysis of the data.

There are many other benefits than can be found at the Goddard Spaceflight Institute (GSFC) primary CDF website at CDF.GSFC.NASA.gov. When considering a data file format specification such as CDF it is important to also consider the impact of the disadvantages in making the choice:

1. CDF files are stored as binary data and while the data in uncompressed CDF files can be read directly by user developed software in general proper access to the data is best done through the available CDF Software Suite directly integrated into a user’s program or accessed via Commercial software.
2. CDF itself has the capability to store multidimensional data but all of the data within the array must be of the same type. CDF does not have the capability to store “objects” or otherwise records comprised from a heterogenous amalgamation of data with varying data type such as one might find in a Jason (ref) XML stream.
3. CDF itself has many software interface readers but a CDF file itself cannot be read by other common software program such as Microsoft Excel which many data analysts find useful especially in doing validation between various data records or just doing simple plotting without having to purchase a large-scale commercial software package such as IDL.

### Energetic Particle, Composition, and Thermal Plasma Suite (ECT-) (Spence et al. 2013)

#### HOPE Level 2 Processing Algorithms

HOPE data are affected by changes in on-board energy and angular bins, both over the course of the mission and within an orbit. These are described in Skoug et al. (in prep).

HOPE fluxes incorporate a time-varying efficiency correction. This algorithm, and other details of HOPE processing, are detailed in Voskresenskaya et al. (in prep). For each of the five sensor heads (pixels) and 72 energy channels, an absolute efficiency is calculated as (coincidences ∗ coincidences)/(starts ∗ stops) and normalized to January 2013, with the switch to the final 72-bin energy assignments on board occurred. This value is calculated hourly by summing each of the counts for the hour; if an hour contains less than 3600 coincidence counts, the time window is expanded until the threshold is reached, and the same relative efficiency value used for all times within the window. Data gathered for $\text{L}<2.5$ are excluded. Earlier releases of the data used a variant of this algorithm; the earliest releases contained no time-varying correction.

#### HOPE Level 3 Processing Algorithms

HOPE level 3 files are calculated from the level 1 (counts) files and an intermediate pitch angle tags product.

Time tags from level 1, which has a single tag for the entire spin and all energy sweeps, are converted to a single unique tag for each sector of the spin, and each energy value, time-resolving the energy sweep. The spacecraft spin is broken into sectors by HOPE, and during each sector a complete energy sweep is made. This means different energies are measured at different times, and thus slightly different spin phases and look directions. EMFISIS Level 2 data are used to find the magnetic field for each of these timestamps, with the field interpolated to the timestamp from available field data using the SLERP approach as implemented by LANLGeoMag (Henderson et al. 2018). The angle between the field and the HOPE look direction (rotated into the same frame as the magnetic field using SPICE) is calculated for each record, detector, sector, and energy, and recorded as the pitch angle (0–180 degrees) in the “tags” file. An orthogonal gyro angle (0–360 degrees) is also calculated. Zero gyroangle is defined as the direction of the cross product of the magnetic field direction and the spacecraft spin axis. Before EMFISIS level 2 files were available, EMFISIS quicklook files were used, but they have not been used for the final archive.

From the level 1 counts and the pitch angle tags, a general binning code creates binned level 3 files. This code sums counts into 2D array (for each time) by pitch angle and gyrophase, also tracking total number of samples in each bin. These arrays are treated identically to the 2D array, by detector number and sector. Thus, the same code that calibrates counts to fluxes (count rates, uncertainties) for level 2 is used to calculate fluxes in level 3. For level 3 pitch angle files, a single gyrophase bin is used (and removed on output), with eleven pitch angle bins: nine bins of 18 degrees, and half-width bins for 0–9 and 171–180 degrees, to provide higher resolution at the loss cone. The same code and inputs are used to produce files containing 5 pitch angle and 8 gyroangles as inputs to moment calculations.

#### REPT Processing Algorithms

The REPT processing algorithms are described in Baker et al. (2021). A summary of the basics of the algorithm proceeds in the following sequence. A particle enters the sensor stack and deposits charge on the silicon into Charge Sensitive Amplifiers (CSA). The CSA collects the charge (or current) pulses from each detector in the stack and then generates a classification of the pulse using Pulse Height Analysis (PHA) techniques. The particles are classified by type and energy range by comparing the PHA values from the individual detectors and PHA sums of select detectors against sets of defined energy bounds, each set defines the energy and species of each particle. The identified bounding set is then used to increment the counting for that particular species/energy combination. The energy bins are accumulated over each spacecraft spin into sectors (36/spin). Counts are then reported as a telemetry record one per spin providing fine pitch angle discrimination. The collective data for each spin is then analyzed by energy spectra per pitch angle.

#### Combined Electron Product

A combined electron product, using all ECT sensors (HOPE, magEIS, REPT), is described by Boyd et al. (2021).

#### Magnetic Ephemeris variables

To provide easy context to the scientific observations, certain quantities from the magnetic ephemeris files (Reeves et al. in prep) are added to all ECT data files. These are interpolated from the one-minute MagEphem files to the same timestamps as the ECT data. This postprocessing step is applied after the generation of the L2 and L3 files with instrument-specific code, using a single generic code. Included quantities are MLT, Roederer L*, model magnetic field at the spacecraft, McIlwain L, model equatorial field, and spacecraft position in geographic coordinates. All are using the OP77Q model.

### Electric and Magnetic Field Instrument Suite and Integrated Science (EMFISIS)

The EMFISIS instruments (Kletzing et al. 2013) have operated as planned throughout the mission with essentially no changes. A few parameters have been adjusted:

- From Oct 2012–Dec 2012, the length of the electric field booms was increasing, so the parameter used for calculating the electric field was adjusted to provide the correct length as the booms were extended These factors are incorporated into the EMFISIS data products.
- After monitoring the response to large amplitude signals, the built-in attenuator was switched on continually after early 2013 to ensure minimal clipping of signals. This cut is 15 dB and is included in the physical units for EMFISIS data products.
- Approximately halfway through the mission the threshold for change magnetometer ranges was lowered by about 500 nT to ensure correct switching as the spacecraft moved outbound. Because of the rapid mothing of the spacecraft, this change is essentially unnoticeable, moving the location of the change outbound by a fraction of an Earth radius. Indeed, after the change no date users ever noticed!

Beyond these operational changes in the instruments, EMFISIS steadily revised software to correct for the usual coding and calibration errors. For L2 and L3 products there have essentially no change since the second quarter of 2021.

The EMFISIS L4 density product has remained unchanged in form, but due to the need for human intervention to ensure accuracy, the data set is not 100% complete, but is complete at a level of great use to the community.

The L4 wave-normal analysis (WNA) project (described in the EMFISIS post-flight instrument paper) has been the subject of intensive work to improve the electric field accuracy by employing a model of the sheath impedance to the plasma to get correct amplitudes and phases. This effort has been quite successful and provides one of the most accurate sets of 3D electric and magnetic field wave products in terms of parameters such as Poynting flux, ellipticity, polarization, etc.

Some data products produced by EMFISIS were not originally planned for, but were developed because of their utility. These include records of thruster firings, spacecraft charging events, and axial boom shadowing. EMFISIS also developed a data product to provide a set of spacecraft housekeeping data so that instruments could understand housekeeping events which might affect their operation.

### Electric Fields and Waves Suite (EFW)

A description of the instrument and science operations for the Electric Fields and Waves (EFW) instrument is provided by Wygant et al. (2013). The primary activities of the EFW Science Operating Center (SOC) – divided between the University of Minnesota and the University of California Berkeley – included data processing, instrument operation and commanding, scheduling of sensor diagnostic tests, and the collection and telemetry of burst data including support of collaborative campaigns with other missions.

Here we discuss the EFW data processing chain leading to the production of publicly available data products, and the operation of the burst 1 instrument. Further details are available in Breneman et al. (2022).

This section is an overview of the EFW data processing chain from raw telemetry (level 0) files to fully calibrated, publicly available level 3 files. On a near daily basis UCB SOC received raw telemetry files from the Mission Operating Center (MOC) at Johns Hopkins University Applied Physics Laboratory. These were decommutated and turned into time-tagged but un-calibrated (L1 ADC counts) science and housekeeping data quantities. These files were then transferred to UMN SOC where they underwent further calibration. This included the application of a rough calibration to attain physical units (such as mV/m) used to produce the daily survey quick look plots available at http://rbsp.space.umn.edu/survey/. In a few days’ time, after official ephemeris data and (roughly calibrated) EMFISIS magnetometer data became available, the quick look plots were updated to include the more accurate spin-fit survey electric fields. In addition, calibrated L2 files were developed, and these included quantities such as spin cadence (spin-fit) electric fields in modified GSE (mGSE) coordinates (see Breneman et al. 2022), survey cadence (16 or 32 s/sec) electric-fields in mGSE, probe potentials, and estimates of plasma density. Finally, in the following weeks or months, L3 data containing the best calibration available were produced as ISTP-compliant CDF files. These files, available at CDAWeb, represent the best possible EFW calibrated data and are recommended for public use.

### Radiation Belt Storm Probes Ion Composition Experiment Science Operations

**Fig. 1:**
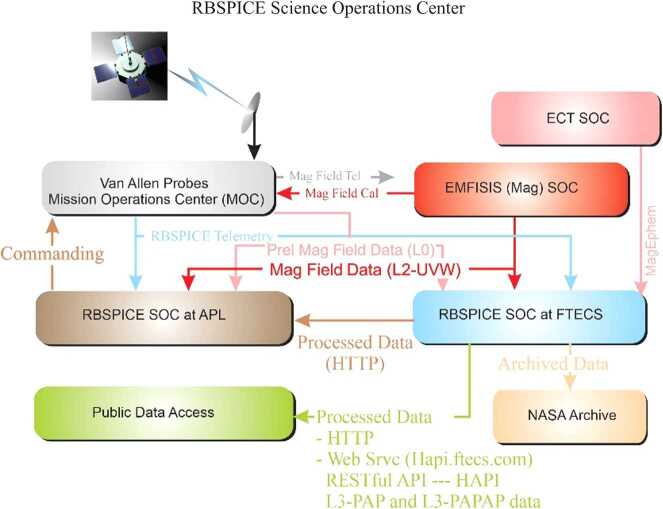
RBSPICE Data Flow Schematic. Figure derived from Mitchell et al. (2013) and updated with final implemented information

The RBSPICE Science Operations Center (SOC) as described by Mitchell et al. (2013) was developed over the course of five years prior to launch. Development and enhancement of the operational and scientific software continued throughout the duration of the seven-year mission. This section the changes and enhancements to the RBSPICE SOC and data as compared to Mitchell et al. (2013). Figure 1 presents the final data flow schematic as implemented by the RBSPICE SOC, located at Fundamental Technologies, LLC (FTECS) in Lawrence, KS, and the RBSPICE SOC located at JHUAPL in Laurel, MD. Pre-release Magnetic field data (EMFISIS-L0) was included to allow the RBSPICE SOC to create preliminary pitch angles for analysis in the MIDL software. Enhancements to the external interfaces from FTECS included the development of a RESTful API based upon the Heliophysics Application Programmer’s Interface (HAPI – see Vandergriff et al. 2019) which allows for streaming of RBSPICE data using a JSON object specification.

#### Summary of RBSPICE Data Pipeline and Products

The RBSPICE data processing pipeline was architected and designed using the Unified Modeling Language (UML – Rumbaugh et al. 2004; Booch et al. 1999). It was implemented in Microsoft C# (Hejlsberg et al. 2006) to run on Microsoft Windows 8.1 with final production occurring on Microsoft Windows 10. The production systems were based upon software and systems used for production of the Cassini Magnetospheric Imaging Instrument (MIMI) data (Krimigis et al. 2005) although significant modifications and enhancements were made to the overall software systems with a design toward utility and generalization instead of high-speed performance.

The RBSPICE data production pipeline was developed as a series of segments based upon NASA Data Level definitions (see the Appendix) (CSV = Comma Separated Value, CDF = Common Data Format (Kessel et al. 1995)). Production of each NASA data level in the RBSPICE SOC occurred as a set of dependent steps with all data products for any particular day being generated for each production segment (NASA data level). Enhancement to the L3 data production included two additional products using team-defined binning algorithms. The primary L3 data product includes the L2 differential flux along with calculated pitch and phase angles for each record for each telescope. Additionally, magnetic ephemeris coordinate information is included which was taken directly from the ECT Magnet Ephemeris (MagEphem) data product (Reeves et al. in prep). See Table 1 for details on RBSPICE data products time resolution, data formats, and primary data units.

**Table 1:** List of RBSPICE data products per NASA Data Level including time resolution, data formats, and primary data units

Data Level	Description	Time resolution	Format(s)	Units
L0	Repackaged CCSDS PTP (Payload Telemetry Packet) records	Sector based	CSV, CDF	[# (counts)],
L1	Rate data in instrument units	Sector	CSV, CDF	[#/sec]
L2	Differential flux in physical units	Sector	CSV, CDF	[#/(cm^2^∗sec∗str∗MeV)]^a^
L3	Copy of L2 Differential flux packaged with: Magnetic field as pitch angles and specific Ephemeris data	Sector	CDF	[#/(cm^2^∗sec∗str∗MeV)]
L3-PAP^b^	Differential flux binned into Pitch Angle bins	Spin	CDF	[#/(cm^2^∗sec∗str∗MeV)]
L3-PAPAP^b^	Differential flux binned into Pitch and Phase angle bins	Spin	CDF	[#/(cm^2^∗sec∗str∗MeV)]
L4	Phase Space Density (PSD)^c^	Spin Averaged	CDF	[#/(km^6^/sec^3^)]

^a^Units defined in COSPAR ISTP Panel on Radiation Belt Environmental Modeling (PRBEM) standards (Bourdarie et al. 2012)

^b^PAP and PAPAP designations are not part of the NASA Data level specification but are included since they represent additional RBSPICE Level 3 data products have alternative data organization strategies

^c^L4 PSD was an originally proposed data product but due to limitation of resources is not currently planned for production

The L3 Pitch Angle and Pressures (L3-PAP) contains L2 differential flux binned by pitch angle, species specific perpendicular and parallel partial pressures, OMNI flux, total intensity, and the species specific partial density. Pressures and density calculations include binned flux for a limited set of energy channels chosen as reliable and uncontaminated by the RBSPICE instrument/science team. The L3 products called the Pitch Angle, Phase Angle, and Pressures (L3-PAPAP) contains L2 differential flux binned by pitch and phase angles. Phase angles are calculated using the Solar Magnetospheric (SM) reference frame with the zero-degree phase toward the Sun ($\hat{x}_{SM}$), and the $90^{\circ}$ phase in the $- \hat{y}_{SM}$ direction. L3-PAPAP includes the calculation of species-specific pressures, OMNI flux, intensity, and density as in the L3-PAP files. See Table 2 for details on each data category identifying the data sources, units, access, and overall mission data volume. The RBSPICE instruments were capable of distinguishing between electrons and individual ion species, specifically protons, helium, and oxygen – for further instrument details see Gkioulidou et al. (2022).

**Table 2:** List of RBSPICE data products by NASA Data Level with data source, accessibility, and total mission data volume

Data category	Data source	Measurement type/units	Publication/access level	Mission data volume
MOC Data Products – not instrument specific	MOC	NA	RBSPICE team only	$\sim419~\text{GB}$ – A $\sim407~\text{GB}$ – B
RBSPICE Instrument Data (telemetry/Level 0)	RBSPICE SOC	Counts [#]	RBSPICE team only	$\sim514~\text{GB}$ – A $\sim500~\text{GB}$ – B
RBSPICE Level 1 Data	RBSPICE SOC	Rate [#/sec]	RBSPICE team and Archive systems	$\sim1.93~\text{TB}$ – A $\sim1.87~\text{TB}$ – B
RBSPICE Level 2 Data	RBSPICE SOC	Flux [#/(sec∗sr∗cm^2^∗MeV)]	RBSPICE team and Archive systems	$\sim2.75~\text{TB}$ – A $\sim2.64~\text{TB}$ – B
RBSPICE Level 3 Data	RBSPICE SOC	Flux and Pitch angles [#/(sec∗sr∗cm^2^∗MeV)]	General Public	$\sim1.43~\text{TB}$ – A $\sim1.38~\text{TB}$ – B
RBSPICE Level 3 PAP data	RBSPICE SOC	Binned Flux by Pitch Angle [#/(sec∗sr∗cm^2^∗MeV)]	General Public	$\sim230~\text{GB}$ – A $\sim224~\text{GB}$ – B
RBSPICE Level 3 PAPAP data	RBSPICE SOC	Binned Flux by Pitch/Phase [#/(sec∗sr∗cm^2^∗MeV)]	General Public	$\sim900~\text{GB}$ – A $\sim840~\text{GB}$ – B
RBSPICE Level 4 data	RBSPICE SOC	Binned Phase Space Density (s^3^/km^6^)	General Public	∼TBD ∼TBD

#### Time System Specifications

The RBSPICE time system utilized the NASA Navigation and Ancillary Information Facility (NAIF) SPICE software system (Acton 1996; Acton et al. 2017) to convert spacecraft time (SCLOCK) into the J2000 Ephemeris Time system (ET) (Fukushima 1995). The MOC was responsible for the production of SPICE kernels maintaining the temporal map between SCLOCK and ET (J2000 epoch). All spacecraft clock event resets were handled by the MOC without creating new SCLOCK partitions.

MOC generate data files were produced for each SCLOCK day (86400 SCLOCK ticks). The first SCLOCK day was created in synch with the UTC Day of launch. Each SOC produced UTC Day files for each data product. This required correct handling of input telemetry files realizing that any particular day of telemetry might include data from as many as three different UTC days. The RBSPICE SOC system created a database map of the SCLOCK to UTC start and stop times for each MOC telemetry file. This allowed for a fast query to find telemetry files containing data for any particular UTC Day.

#### Telemetry Processing and Data Production

The RBSPICE Level 0 data contains 33 individual data products, see Table 3 for a listing of the primary counting data products together with the required ancillary data products used for production. Each product file contains an unpacked copy of the RBSPICE telemetry file decoding the CCSDS Payload Telemetry Packet (PTP) (Packet Telemetry 2000) records into count and support data. The only time field provided by each PTP is the spacecraft SCLOCK and the internal RBSPICE flight software derived time fields.

**Table 3:** RBSPICE Data Products catalogue of primary counting products along with required ancillary data products showing species, number of energy channels, and the type of data generated at each NASA data level

Product	Species	Energy bins	L0 data type	L1 data type	L2 data type	L3 data type	L4 data type
Electron Energy Mode Basic Data	e^−^	NA	Counts	Rates			
Ion Energy Mode Basic Data	Ions	NA	Counts	Rates			
Ion Species Mode Basic Data	Ions	NA	Counts	Rates			
Low Energy Resolution High Time Resolution Electron Species Data^1^	e^−^	14	Counts	Spectra	Spectra Flux	PAD, Aggregates	PSD, 2nd, 3rd Adiabat,
High Energy Resolution Low Time Resolution Electron Species Data^1^	e^−^	64	Counts	Spectra	Spectra Flux	PAD, Aggregates	PSD, 2nd, 3rd Adiabat,
High Energy Resolution Low Time Resolution Ion Species Data^1^	Ions	64	Counts	Spectra	Spectra Flux	PAD, Aggregates	PSD, 2nd, 3rd Adiabat,
High Energy Resolution Low Time Resolution TOFxPH Proton Data	H^+^	32	Counts	Spectra	Spectra Flux	PAD, Aggregates	PSD, 2nd, 3rd Adiabat,
TOFxE Proton Data	H^+^	14	Counts	Spectra	Spectra Flux	PAD, Aggregates	PSD, 2nd, 3rd Adiabat,
TOFxE non-Proton Data	$\text{He}^{n+}$, $\text{O}^{n+}$	28	Counts	Spectra	Spectra Flux	PAD, Aggregates	PSD, 2nd, 3rd Adiabat,
Low Energy Resolution High Time Resolution TOFxPH Proton Data	H^+^	10	Counts	Spectra	Spectra Flux	PAD, Aggregates	PSD, 2nd, 3rd Adiabat,
TOFxE Ion Species Data	Ions	64	Counts	Spectra	Spectra Flux	PAD, Aggregates	PSD, 2nd, 3rd Adiabat,
Space Weather Data	All	NA	Counts	Rates	Flux		
Auxiliary Data	NA	NA	Aux data				
Critical Housekeeping Data	NA	NA	HSK				

The produced L0 products include three time fields formats: ET (double precision), SCLOCK (string), and Universal Time Coordinated (UTC-string) using the ISO(T) 8601 Ordinal Time Format Specification [ANSI INCITS 30-1997 (R2008) formatted as “CCYY-DDDTHH:MM:SS.hhh”. CCYY = century and year, DDD = ordinal Day of Year, HH = hour, MM = minute, SS = integer second, and hhh = decimal seconds to milliseconds resolution. SCLOCK values are formatted in NAIF Type 1 SCLOCK format (NASA NAIF SPICE 2010) as [part/ticks:fine] where part = integer partition (always 1), ticks = major ticks ($\sim1~\text{second}$), and fine = minor ticks of the spacecraft time system in $2^{-16}$ increments.

The following sections provide updates to the algorithms used in the creation of the Level 0 Count Files, the Level 1 Rate files, and the Level 2 Intensity (flux) files. Subsequent sections provide the detailed algorithms used in the creation of the Level 3 Pitch Angle files, the Level 3 PAP files, and the Level 3 PAPAP files. A final section discusses the algorithms needed to calculate Level 4 Phase Space Density (PSD) data. Details presented for each of these steps are sufficient in conjunction with the details provided in the original MB-I and the RBSPICE Data Handbook (Manweiler and Zwiener 2019) to allow other software developers to write their own translation workflow.

#### Level 0 Data Product

The Level 0 data products are organized by ephemeris time (ET), spacecraft spin number, and the RBSPICE instrument created virtual sector number with 36 sectors per spin. The starting time of each sector is determined by the RBSPICE flight software coupled with the spacecraft 1 PPS (Pulse Per Second) status record sent to the RBSPICE instrument. The ground software calculates the beginning of each sector based upon the nominal spin period provided by the RBSPICE Auxiliary telemetry record for the current spin/sector using the Spin Duration field. In the situation where either spacecraft goes into eclipse and loses the nominal 1 PPS signal then the RBSPICE flight software utilizes a hard-coded nominal spin period of 12 sec to calculate the duration of each spin and to time tag the beginning of the next spin record.

#### Time Stamp Generation

**Fig. 2:**
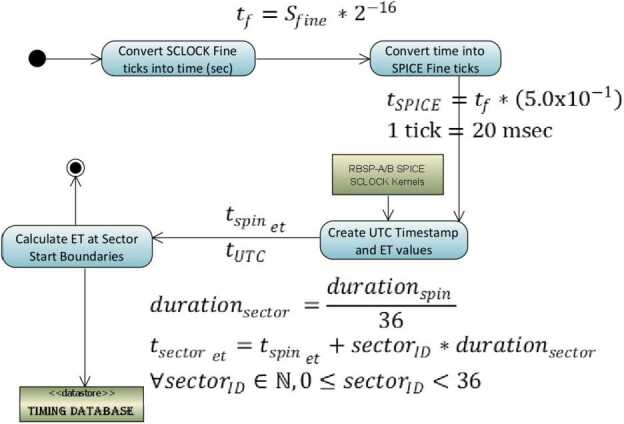
Diagram showing the calculation of timing factors for RBSPICE telemetry

The RBSPICE Auxiliary telemetry (Aux) product is the only component of the received RBSPICE telemetry that provides the ability to create a high time resolution conversion from the full SCLOCK to ET (J2000 epoch). Aux packets are generated by the RBSPICE instrument at the end of each spin and each include a time stamp derived from the timing information provided by the spacecraft 1 PPS (Pulse Per Spin) signal. The SCLOCK value is a four-byte unsigned integer which cycles from 0 to ($2^{32}-1$). The Fine SCLOCK value is a two-byte unsigned integer number which cycles from 0 to ($2^{16}-1$) and is in units of ($1/2^{16}$) SCLOCK ticks. In general, each tick of the SCLOCK is approximately 1 second, although this relationship can drift depending upon the heating and cooling of the spacecraft. The SCLOCK value is not a unique value, but repeats every 136.19 years. A compression of the SCLOCK value from the instrument was necessary when converting into NAIF SCLOCK values since the NAIF Fine specification is in 1/50000 sec units. The $\times323$ telemetry record time stamps are decoded by the RBSPICE SOC software system and the resulting SCLOCK and Fine SCLOCK values are converted into a time stamp using the algorithm in Fig. 2.

#### Duration of Measurement and Start/Stop Times

**Fig. 3:**
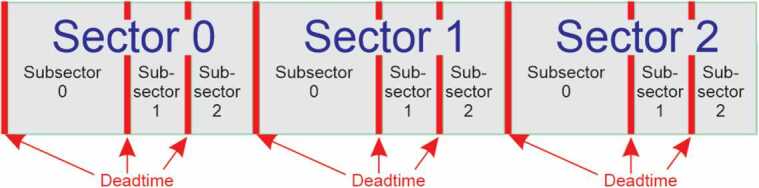
Sector and subsector scheme used by RBSPICE also showing inter-sector and intra-sector dead times

Level 0 processing calculates the duration of each measurement at the same time the sector timestamp is calculated. The duration cannot be simply calculated as the difference between the next sector and current sector start times since the RBSPICE instrument has three possible measurement modes which can be assigned to one of the three available subsector accumulation time periods. Figure 3 displays the sector division into three unequal time sized subsector partitions: $\Delta t_{0} = \frac{1}{2} t_{sect}$; $\Delta t_{1} = \frac{1}{4} t_{sect}$; $\Delta t_{2} = \frac{1}{4} t_{sect}$. The RBSPICE instrument can be commanded to use any measurement mode (electron energy, ion energy, and ion species) in any combination of subsectors, providing the ability to simultaneously measure electrons and ions within a sector or, alternatively, to use a single type of measurement for higher time resolution science. Sector “dead time (dt)”, also shown, occurs at the end of each subsector due to instrument electronic state changes, $\Delta t_{01_{dt}} = 3.94~\text{ms}$; $\Delta t_{12_{dt}} =3.95~\text{ms}$; $\Delta t_{20_{\text{dt}}} =4.04~\text{ms}$. Subsector accumulation time is $\Delta t_{0_{acc}} = \Delta t_{0} - \Delta t_{20_{dt}}$; $\Delta t_{1_{acc}} = \Delta t_{1} - \Delta t_{01_{dt}}$; $\Delta t_{2_{acc}} = \Delta t_{2} - \Delta t_{12_{dt}}$.

**Fig. 4:**
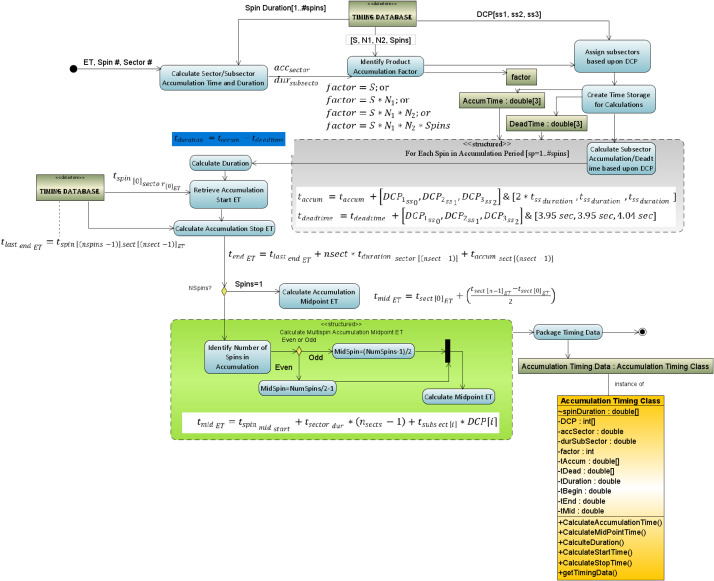
Activity Diagram showing the algorithmic steps in the production of the Level 0 files

**Fig. 5:**
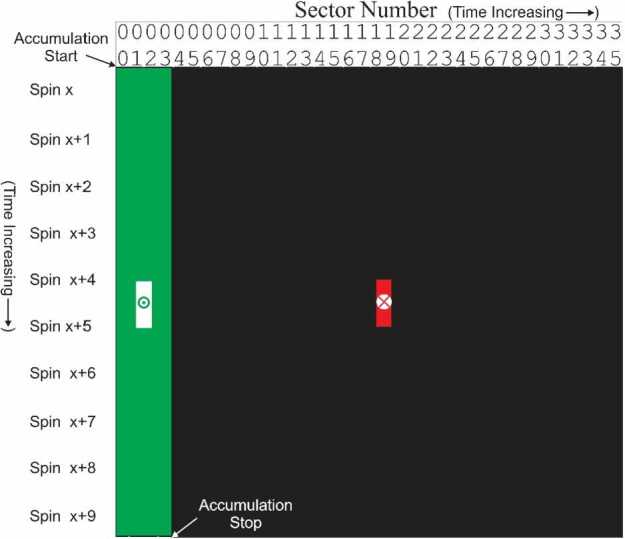
Shows the false (red) and correct (green) midpoint calculations of the midpoint time for the current multi-spin accumulation period over a few sectors

The key values required to properly calculate the measurement duration are found in the Aux telemetry packet: Spin Duration (in seconds), Accumulation Mode Values (S, N1, N2, Spin) and Data Collection Pattern (DCP) – the combination of instrument modes for each subsector. The timing system calculates the duration of the measurement using the algorithm in Fig. 4. The diagram showing the structured activity (green insert box) provides some detail of the calculation of the midpoint time for the accumulation. For single spin accumulations this calculation is very straight forward as the start ET plus half the delta time for the accumulation, ($t_{mid_{ET}} = t_{start_{ET}} + ( t_{end_{ET}} - t_{start_{ET}} )/2$). Multi-spin accumulation involves a more complex calculation, see Fig. 5. In this example, the calculation is done for a starting accumulation in sector 0 and accumulating over 4 sectors and 10 spins, i.e., $S=1$; $N_{1} =2$; $N_{2} =2$; $Spin_{j} =10$. The sectors involved in the measurement are identified in the table as green with a white square in the middle. A “false” midpoint time is calculated using the simple algorithm $t_{mid_{ET}} = t_{start_{ET}} + ( t_{end_{ET}} - t_{start_{ET}} )/2$ as indicated with the “$x$” in the red square outside the actual accumulation time.

The correctly calculated midpoint is shown as the bullseye in the middle of the two white squares. Even this calculation requires attention because the two white squares in the example are still one full spin apart. if the number of spins used in the accumulation is even then the midpoint time is the end of the first of the two white squares but if the number of spins used in the accumulation is odd then there is only a single sector in the white square so the midpoint is halfway between the start and stop of that sector.

The rest of the RBSPICE Level 0 data product production is thoroughly described in the online version of the RBSPICE Data Handbook (Manweiler and Zwiener 2019).

#### Level 1 Processing Algorithms

Level 1 processing is done by converting Level 0 count data into Level 1 rate data as a series of algorithmic steps for which the critical component is the calculation of the Rate-in versus the Rate-out ($R_{in}$ *vs* $R_{out}$) algorithm. This is necessary since the instrument electronics has a maximum clock cycle limiting the highest rates observable before becoming saturated as well as accounting for lost particle events during instrument deadtime. Table 4 presents the fields and their definitions, type, and default values that are used in the subsequent $R_{in}$ *vs* $R_{out}$ formula.

**Table 4:** $R_{in}$ *vs* $R_{out}R_{in}$ *vs* $R_{out}$ variable names, descriptions, variable type, and encoded values

Name	Description	Type	Value(s)	
Max_IDLE_	Maximum number of 100 ns intervals for which data can be accumulated	UInt32	$\frac{\Delta t}{10^{-7}}$	
Clk_Period_	Number of nanoseconds in the RBSPICE DPU clock period	UInt32	100	
ST_Dead_	Start counter dead time due to synchronization logic	UInt32	2	
SP_Dead_	Stop counter dead time due to synchronization logic	UInt32	2	
SP_Veto_	Interval in which additional stop pulses cause the event to be discarded	UInt32	2	
RDT_Veto_	Interval for inhibiting start and stop counter during chip TOF reset	UInt32	1	
PKD_Reset_	Interval for resetting the peak detector	UInt32	4	
PUR_Veto_	Interval during which a second SSD pulse causes event to be discarded	UInt32	7	
TOFxE_PUR_Veto_	Interval during which a second SSD pulse causes event to be discarded (*changed in software configuration file for TOFxE only*)	UInt32	24	
K_1E_E_	Correction constant term for valid TOFxE events	Float	0.3	
K_1E_PH_	First order correction constant term for valid TOFxPH events	Float	0.15	
K_2E_PH_	Second order correction constant term for valid TOFxPH events	Float	0.15	
ST_MISS_	The number of FPGA clock cycles are missed each sectorCode variable names: _tofxph_RvsR_EFact or _tofxe_RvsR_Efact	UInt32	2	
Cssd	FPGA clock ticks or the required value to reproduce MHR from FPGA, based upon the IBSR record only	UInt32	2	
C_phSC_	Represents the factor for which PH counts miss from the start0 counts		Float	$C_{phA}=0.860$ $C_{phB}=0.775$

#### $R_{in}$*vs*$R_{out}$ Algorithm and Formula for Specific Data Products

**Fig. 6:**
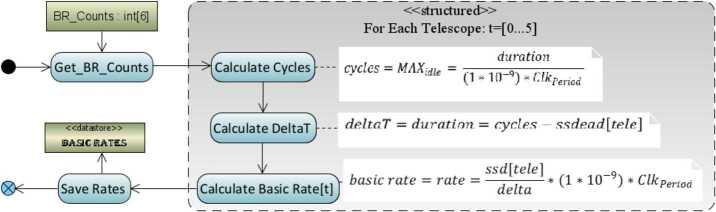
Algorithmic diagram displaying the conversion of basic counters into basic rates

***Basic Rates: EBR (APID: x312), IBR (APID: x313), and ISBR (APID: x315)*** Basic rate telemetry includes the measured counts (SSD), dead time correction values (SSDDead) per telescope, and the calculated duration of the accumulation. These values are converted to a rate value using the algorithm in Fig. 6.

#### Energy Rates

**Fig. 7:**
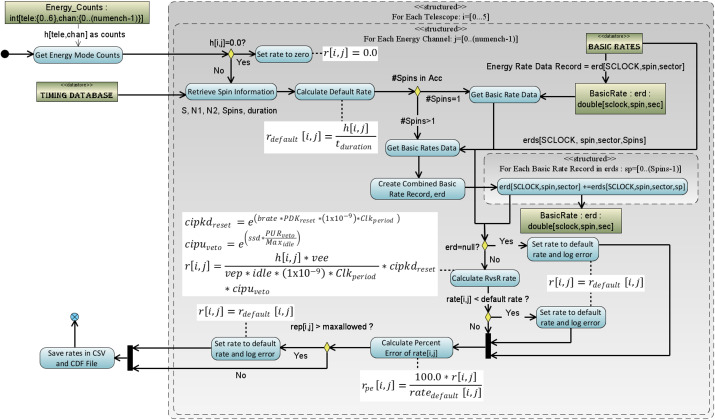
Algorithmic diagram displaying the conversion of Energy Mode counters into Energy rates

The conversion algorithm of the counts obtained for the following energy mode products, ESRLEHT (APID: x317), ISRHELT (APID: x318), and ESRHELT (APID: x319), requires an understanding of the spin information (APID: x323) and the $R_{in}$ *vs* $R_{out}$ corrected basic rate data (EBR for ESRLEHT and ESRHELT, IBR for ISRHELT) to calculate the rate. For purposes of this algorithm, the count values in the telemetry are referenced as $h_{ij}$ where $i$ refers to the telescope number and $j$ refers to the energy channel of the measurement. Figure 7 shows the algorithm used in the RBSPICE SOC software for each telescope and each energy channel. The figure includes the formulas used in the calculations.

#### Species TOFxPH Rates

**Fig. 8:**
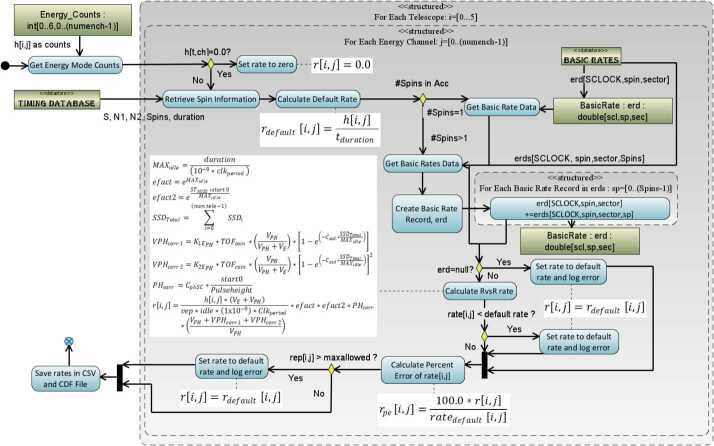
Algorithmic diagram displaying the conversion of TOFxPH Species Mode counters into TOFxPH rates

Figure 8 displays the algorithm used in the conversion of the species mode TOFxPH measurements for products TOFxPHHLEHT (APID: x31D) and TOFxPHHHELT (APID: x31E) which follows the algorithm for the calculation of Energy Rates (see Fig. 7). The key difference in the diagram is the use of the corrected Ion Species Basic Rates (ISBR – APID: x315) and differences in the formula used in the $R_{in}$ *vs* $R_{out}$ calculation.

#### Species TOFxE Rates

**Fig. 9:**
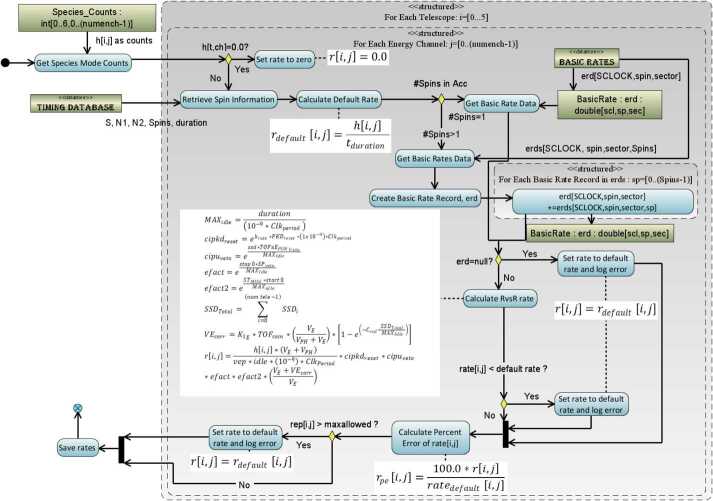
Algorithmic diagram displaying the conversion of TOFxE Species Mode counters into TOFxE rates

Figure 9 displays the algorithm used in the conversion of the species mode TOFxE measurements for products TOFxEIon (APID: x31A), TOFxEH (APID: x31B), and TOFxEnonH (APID: x31C) follows a similar algorithm as for Species TOFxPH rates (see Fig. 8). The key difference in the diagram is the formula used in the $R_{in}$ *vs* $R_{out}$ calculation.

#### Error Calculations for Rate Files

**Fig. 10:**
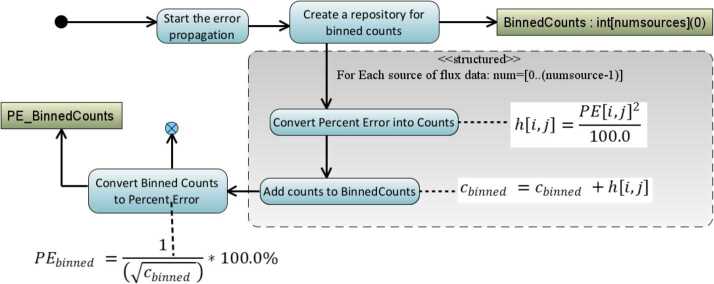
Error propagation algorithm used in later data production especially for the Level 3 PAP and PAPAP files where flux is binned and the errors for any particular bin must be carefully calculated

As counts are converted into rates, the Level 1 files capture the statistical Poisson error for the purposes of error propagation in later data levels. Additionally, since we are keeping track of the percent error and including the errors in higher level data products, we have the ability to easily propagate the errors when we do various integration or telescope combination activities in the level 3 data products, see discussion of errors in the Level 3 PAP/PAPAP sections and also Fig. 10 for the basic error propagation algorithm used in the RBSPICE production system.

#### Level 2 Processing Algorithms

The primary activity in processing the Level 1 data into Level 2 data is to convert the rate data into particle intensity (flux) data. This is done in a series of algorithmic steps in which the Level 1 rate data is read into memory, the calibration data for the SC and product are loaded, the intensities are calculated, and the intensities are then written to a Level 2 file. Additional fields are added to the Level 2 file in order to partially fulfill the standards defined by the Panel on Radiation Belt Environmental Modeling (PRBEM: COSPAR ISTP PRBEM Committee 2010).

#### Calculation of Intensities (Differential Flux)

Conversion of RBSPICE data into differential flux requires knowledge of the channel and product specific RBSPICE calibration factors. The calibration data can be found on the RBSPICE website at the following locations: http://rbspice.ftecs.com/RBSPICEA_Calibration.html and http://rbspice.ftecs.com/RBSPICEB_Calibration.html or archived at the CDAWeb RBSPICE archive. Note that the reference table in the calibration files of TOF-trigger_ion E is referring to the RBSPICE TOFxE_Ion data products.

The data is organized by product type and contains the necessary information needed to convert RBSPICE rates into differential flux. The calibration data fields are fully described in the RBSPICE Data Handbook. Rates are converted into Intensities using the following equation, $flux[tele,enchan]= \frac{rate[tele,enchan]}{\left ( E_{High} - E_{Low} \right ) * G_{size} *eff}$. The value of the geometrical factor, $G_{size}$, is based upon the current pixel value (small or large) identified in the Aux data packet for the current spin/sector and ET combination. The final CDF variable that is created to contain the intensities is a two-dimensional variable of type Double (or Double Precision) and sized as $FxDU[tele,enchan]$ so that it contains the data for each telescope and energy channel combination.

#### Ion Species Mode Flux Data (ISRHELT)

While the calibration of the rate and flux data measurements for the Ion Species Rates High Energy Resolution Low Time Resolution (ISRHELT) data product is very well understood the measurement is not itself very specific as to species. In order to use the ISRHELT data it is necessary to have independent knowledge of which species dominates the measurements. Otherwise, it is difficult to draw scientific conclusions using this data product. Any research that utilizes this particular data product should at the very least do a relative comparison to the equivalent ECT-MagEIS measurements before using this data to make scientific conclusions.

#### RBSPICE Background Contamination

The current data files produced by the RBSPICE SOC are not background corrected for contamination. Work is ongoing within the RBSPICE team to correct for these issues but at the time of this writing the rates are still potentially contaminated with accidentals (mostly during perigee) and other background rate contamination issues. *The reader is strongly encouraged to reach out to members of the RBSPICE team prior to doing significant scientific activity in order to avoid utilization of contaminated data and deriving erroneous results*. The two specific products that are most likely contaminated with background or accidentals are a varying set of the TOFxPH proton lowest energy channels and all of the TOFxPH oxygen channels. At some point, the RBSPICE SOC will reprocess the data and at that point when background rates dominate over foreground rates on a channel-by-channel basis then the channel specific data quality flags contained within the CDF files for each flux variable will be properly tagged with a value indicating data contamination. As of the writing of this manuscript the TOFxPH oxygen data have all data records tagged as contaminated. Work is ongoing to attempt to eliminate the background from the data.

#### Level 3 Processing Algorithms

Processing Level 2 data into Level 3 data requires the calculation of the look direction, pitch angle, and phase angle of each telescope, using the measured magnetic field received from the EMFISIS instrument as well as loading of ancillary data from the ECT Magnetic Ephemeris data files.

#### EMFISIS Magnetic Field Data

EMFISIS Level 2 UVW magnetic field data files were used to calculate the RBSPICE pitch angles. These files contain data sampled at 60 Hz with over 5 million samples per data file. In order to reduce memory utilization and processing requirements, these files were deprecated by a specific programmable number prior to pitch angle calculations. The final mission wide deprecation factor was set to 8 representing a signal frequency of 7.5 Hz which results in approximately 2–3 magnetic field measurements per RBSPICE sector. No other filtering of the EMFISIS data was utilized during the deprecation stage, although it is noted that data records tagged as ‘**bad**’ were not included.

#### ECT Magnetic Ephemeris Data

Additional fields loaded in the RBSPICE Level 3 CDF files were derived from ECT Magnetic Ephemeris data files. The definitive Olsen-Pfitzer 1977 quiet time data were used as the source. Specific data fields used were deemed necessary and pertinent to provide for a full scientific understanding of the RBSPICE energetic particle data: ${L}_{dipole}$, ${L}^{*}$, ${L}_{eq}$, I (2nd adiabatic moment – single value and pitch angle dependent array), K (3rd adiabatic moment – single value and pitch angle dependent array), and Magnetic Local Time (MLT).

#### Calculation of Particle Flow Direction

**Fig. 11:**
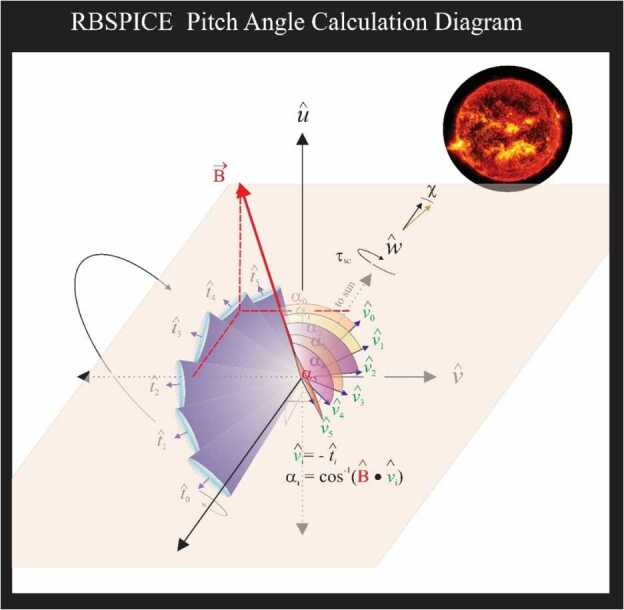
Geometry of the calculation of the RBSPICE Pitch Angles based upon the particle flow velocities for each telescope in the spacecraft UVW reference frame

The particle flow direction has been added to the RBSPICE Level 3 files since file version x.1.10. The calculation of particle flow direction, $\hat{v}_{0},\dots,\hat{v}_{5}$ in Fig. 11, uses the definitive SPICE CK, FK, and IK kernels for each spacecraft at the time of the observations. The calculation utilizes the NAIF SPICE function $\mathrm{pxform}\_c\left ( f,t,tmatrix \right ){:}\ i= \{ n \mid n \in \mathbb{N}, 0\leq n\leq 5\}$. The variable $f$ represents the “From” reference frame and is the RBSPICE telescope reference frame ($RBSP\{A/B\}\_RBSPICE\_ T_{i} \}$, e.g. $RBSPB\_RBSPICE\_ T_{3}$ represents RBSPICE telescope 3 of spacecraft B. The variable $t$ represents the “To” reference frame and is the Spacecraft *UVW* reference frame. The RBSPICE telescope and spacecraft UVW reference frames are defined in the Van Allen Probes SPICE frame kernels: rbspa_vxxx.tf and rbspb_vxxx.tf where “xxx” is the highest version number.

The particle flow direction unit vector is then calculated as the negative or reverse of the telescope boresight unit vector transformed into the *UVW* reference frame, e.g., $\hat{v}_{i} =- \hat{T}_{i_{SM}}$. Any exceptions occurring during this transformation results in the particle flow direction unit vector set as $\hat{v}_{i} =(0.0,0.0,0.0)$ representing an unknown direction.

#### Calculation of Pitch Angles

Figure 11 also displays the geometry used in the calculation of the RBSPICE pitch angle for each of the instruments six telescopes. The overall orientation of the diagram is such that the spacecraft $\hat{w}$-axis points generally toward the sun. The spacecraft rotation around the $\hat{w} $-axis is also shown and the fan of six RBSPICE telescopes allow for an almost $4p$ steradian view of the sky for each spacecraft spin period: $\tau _{SC} \cong 10.9~\text{sec}$. The conical elements of the figure display the telescope look direction unit vectors, $\hat{t}_{i}$, centered on the aperture for each telescope as they are mounted on the spacecraft. The particle velocity unit vectors (or particle flow direction) are also shown in the diagram along with the representation of the pitch angles as the angle between the velocity unit vectors and the observed magnetic field unit vectors. The deprecated 7.5 Hz magnetic field signal results in approximately 2–3 magnetic field vectors occurring in the RBSPICE sector ($\sim0.3~\text{sec}$) time window.

**Fig. 12:**
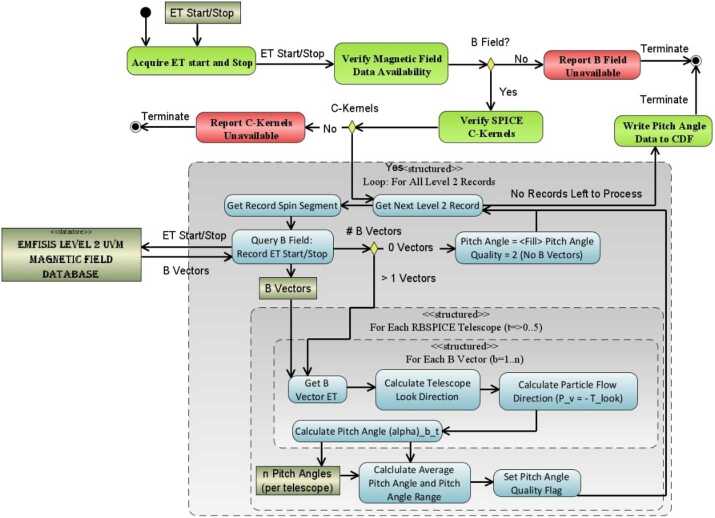
Algorithmic description of the calculation of the RBSPICE Pitch Angles

Algorithmically, a pitch angle is calculated for each of the magnetic vectors that exist within the accumulation period. The final pitch angle is the average of the calculated pitch angles and the deviation between all pitch angles is reported in the CDF variable FxDU_AlphaRange. If the deviation between the calculated pitch angles results in variations that are larger than 1/2 of a sector look direction then the sector pitch angle quality flag is set to a value indicating it is unusable ($\text{AlphaQuality}_{\mathrm{i}}=\{0\text{-Good},1\text{-Bad}\}$) and the pitch angle is set to the CDF Double Precision fill value of $-1.0\times 10^{31}$. Calculation of pitch angles uses the algorithm in Fig. 12.

#### Calculation of Phase Angles

**Fig. 13:**
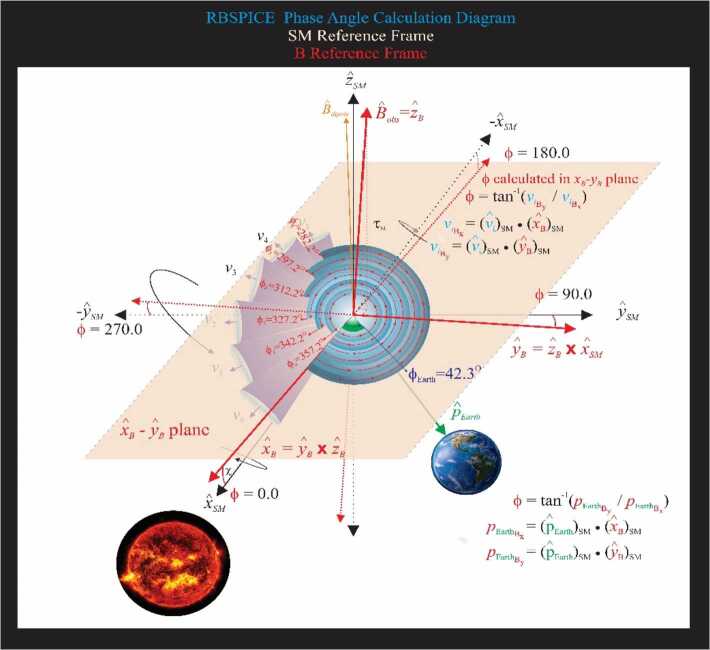
Diagram of the calculation of the phase angle in the SM reference frame. Note that to reduce the complexity of the diagram, the rotation of the spacecraft is shown around the $\hat{x}_{SM}$ axis but the actual rotation of the spacecraft is around the $\hat{w}$ axis of the spacecraft which points approximately along the $\hat{x}_{SM}$ axis

The RBSPICE Level 3 data files, as of file version x.2.z, include a calculation of the phase angle of the RBSPICE telescope with respect to the Solar Magnetospheric (SM) reference frame (Laundal and Richmond 2017). Figure 13 displays the calculation of the phase angles in the $SM$ reference frame. The magnetic field $\hat{x}$–$\hat{y}$ plane is first projected into the SM reference frame and then the phase angles are calculated with respect to the SM coordinate system using the projected vectors of $\vec{B}_{x_{SM}}$ and $\vec{B}_{y_{SM}}$. The orientation of this figure is such that the $\hat{z}$-axis of the SM frame is up (approximately in the direction of $\hat{B}_{dipole}$); the $\hat{x}$-axis is away from the Sun; and the $\hat{y} $-axis completes the orthogonal system. The RBSPICE phase calculation is defined such that the zero-degree phase angle points toward the Sun, i.e., along $+ \hat{x}_{SM}$ and the 90-degree phase angle is in the $+ \hat{y}_{SM}$ direction. As the spacecraft orbits around the Earth, this reference frame always maintains the relationship between the solar drivers of magnetospheric activity and the phase angle of the particle distribution. The figure also shows the particle velocity vectors and the associated acceptance solid angles for each RBSPICE telescope.

**Fig. 14:**
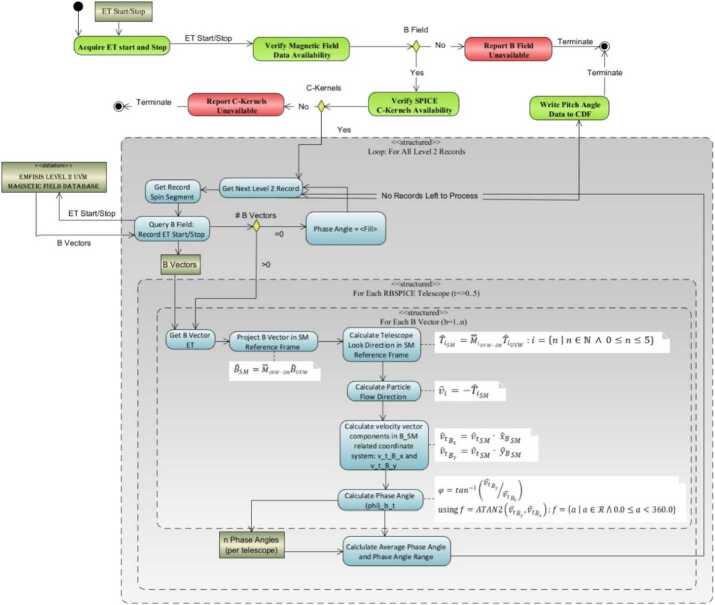
Algorithmic description of the calculation of the RBSPICE Phase Angles

The phase angles are calculated in the $XY_{SM}$ plane and are represented by the blue gradient circles with red lines/arrows starting at the $\hat{x}_{B}$-axis and going to the central point of each cone. An example phase angle is shown with $\text{f}_{0}=357.2^{\circ}$ and each subsequent phase angle $\sim15$ degrees rotated away from the Sun. If the phase angle cannot be calculated then that phase angle is set to the CDF Double Precision fill value of $-1.0\times 10^{31}$. This figure also shows the calculation of the phase angle between the vector that points from the Earth toward the SC and the $\hat{x}_{B}$-axis in the $XY_{SM}$ plane. This allows a phase shift calculation for scientific analysis of Earth centered radial, tangential, and normal particle flow/anisotropies. Figure 14 shows the algorithm used in the calculation of the RBSPICE phase angle.

#### Level 3 Pitch Angle and Pressure (PAP) Processing Algorithms

Level 3 differential flux data is used in the calculation of the Level 3 PAP data products by utilizing the pitch angle data from each telescope and a predefined set of pitch angle bins with centers at 7.5, 20, 30, 40, 50, 60, 70, 80, 90, 100, 110, 120, 130, 140, 150, 160, and 172.5 degrees. Part of the binning of the differential flux provides the ability to calculate partial moments of the distributions. The calculated species-specific moments include the perpendicular and parallel partial particle pressures, density for a select set of energy channels, the omnidirectional differential flux for each energy channel, and fully integrated particle flux over the entire energy range (*Note: proceed with caution as this integrated particle flux includes noisy and background contaminated channels*).

#### Binning of Pitch Angles and Calculation of Aggregate Data

**Fig. 15:**
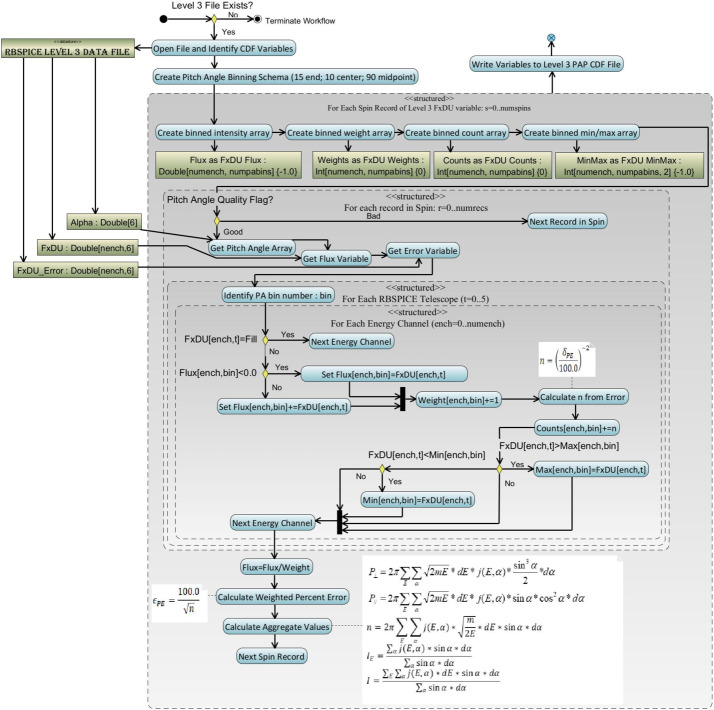
Algorithm for the binning of RBSPICE differential flux and the calculation of moments for the L3 PAP products. With minor modifications this is the same algorithm used in the binning of RBSPICE differential flux and the calculation of moments for the L3

PAP data calculation uses the algorithm shown in Fig. 15. Calculation of the moments is over a specific set of energy channels for which the RBSPICE science team has determined are reasonably reliable. Table 5 presents the energy channels used in moment calculation as a function of data product, energy channel indices (absolute and relative reference channel range with respect to the Level 3 CDF differential flux variable), and the energy channel passband range. Products that are set “none” do not have moments calculated since the specific product has been identified by the RBSPICE team as untrustworthy either in data or in calibration. Untrustworthy data products also have data quality flags set to a value other than 0 = good or 10 = unknown indicating that the data should not be used for science.

**Table 5:** Table showing the specific energy channels used in the calculation of the aggregation (moment) data. Each product shows the absolute channel reference with respect to the Level 3 source data array, the relative channel reference with respect to the energy channels for the specific data product, the mid-point calculated energy passband as well as the full energy passband using the [low, high] values for each passband. Finally, the table shows the mass used in the moment calculation

Product	Energy channels (absolute index)	Energy channels (relative index)	Energy range (KeV) (midpoint passband)	Delta E (KeV)	Energy range (KeV) (low–high passbands)	Delta E (KeV)	Species used for mass calculation
ESRHELT	3–63	3–63	24.1–938.7	914.6	23.38–974.39	951.11	e
ESRLEHT	1–13	1–13	27.4–425.8	398.4	24.7–527.0	502.3	e
TOFxE_H	1–13	1–13	54.7–597.6	542.9	49.0–657.6	608.6	p
TOFxE_He (Pre)	0–8	0–8	65.0–518	453	56.8–584.5	527.7	He
TOFxE_He (Post)	0–10	0–10	65.0–870	805	56.8–982.0	925.2	He
TOFxE_O (Pre)	9–17	0–8	142–1127	985	123.8–1256.0	1132.2	O
TOFxE_O (Post)	11–18	0–7	142–870	728	123.8–998.5	874.7	O
TOFxE_Ion	2–63	2–63	50.6–18525.2	18475	48.4–20000	19952	Ions
TOFxPH_H_LEHT	3–8	0–5	17.4–50.0	32.6 28–100	72	P	
TOFxPH_H_HELT	18–30	7–19	14.8–48.9	34.1	14.1–51.4	37.3	P
TOFxPH_O_LEHT	none	none	NA	NA	NA	NA	O
TOFxPH_O_HELT	none	none	NA	NA	NA	NA	O

*Special note*: As of the writing of this paper the TOFxPH Oxygen observations are deemed unreliable and no aggregate values are calculated within the PAP data files but the data is provided as a product so that the RBSPICE team can update the calibration information and reprocess the data once the causes of the contamination are understood and can be removed resulting in newly calibrated data considered usable for science.

#### Level 3 Pitch Angle, Phase Angle, and Pressure (PAPAP) Processing Algorithms

Level 3 differential flux data is used in the calculation of the Level 3 PAPAP data products by utilizing the pitch angle data from each telescope and a predefined set of pitch angle bins with centers the same as for the Level 3 PAP data product. The predefined set of phase angle bins are calculated in thirty (30) degrees separation with the first center set at zero degrees. Moments are also calculated as with the Level 3 PAP data product and include calculated species specific perpendicular and parallel partial particle pressures, density for a select set of energy channels, the omnidirectional differential flux for each energy channel, and fully integrated particle flux over the entire energy range. An algorithm diagram is not shown for this product as it is almost exactly the same as for the Level 3 PAP algorithm with one change. At the point in which we identify the “PA Bin” number for the record we instead identify the PitchBin and the PhaseBin for the record. The FxDU related variables are expanded with one additional dimension, e.g. FxDU[#energy channels, #Pitch bins, #Phase bins].

#### Level 4 Phase Space Density Data Products

One of the original lofty goals of the RBSPICE science team was to have the capability to generate a standardized Level 4 Phase Space Density (PSD) data product for each of the proton, helium, and oxygen species measured by the RBSPICE instruments. The development of that data product was strongly dependent upon available funding and unfortunately there were not enough resources to support this activity during the mission. The following describes some of the work that was accomplished as part of the initial investigation into the feasibility of the generation of a PSD product and was presented at the in-person Science Working Group (SWG) meetings of the larger Van Allen Probes science teams May 2018 by the lead author.

In a traditional Phase Space Density analysis using magnetospheric plasma and energetic particle data the analysis attempts to blend data observations from particles instruments with a chosen magnetic field model. This method provides a guide to understand the dynamics of the particles through Louisville’s theorem where phase space density organized by an adiabatic invariant coordinate system is unaffected by slow temporal changes in the magnetic field. In the analysis one utilizes the collisional Vlasov equation as the appropriate representative of Louisville’s theorem for plasma physics:

$$ \frac{\partial f}{\partial t} + \vec{v} \cdot \vec{\nabla} f + \frac{q}{m} [ \vec{E} + \left ( \vec{v} \times \vec{B} \right ) ]\cdot\vec{\nabla}_{v} f= \left ( \frac{\partial f}{\partial t} \right )_{coll} $$

As part of the algorithm, it is necessary to convert the observed intensities into a phase space density time series sampling of the distribution function. The conversion from flux, $J(E,\alpha ; \vec{r},t)$ with $[J \left ( E,\alpha ; \vec{r} \right ) ]= \left (\text{cm}^{2}\,\text{sr}\,\text{s}\,\text{MeV}\right )^{-1}$, to phase space density, $f( \mu _{k}, \alpha _{j}; \vec{r},t)$ with $[f \left ( E,\alpha ; \vec{r} \right ) ]= \text{s}^{3} / \text{m}^{6}$, is presented in Schulz and Lanzerotti (1974):

$$ J_{\alpha} \left ( E; \vec{r}, t \right ) = p^{2} f \left ( \vec{v}; \vec{r}, t \right ) \quad\Longrightarrow\quad f \left ( \vec{v}; \vec{r},t \right ) = \frac{J_{\alpha} \left ( E; \vec{r}, t \right )}{p^{2}} $$

and when converting from a velocity space integration into an energy space integration becomes written as:

$$\begin{aligned} f \left ( E, \alpha ; \vec{r},t \right ) = \frac{m^{2} \star J \left ( E, \alpha ; \vec{r},t \right )}{2E} \end{aligned}$$

The analysis is strongly dependent upon binning the data into model dependent adiabatic invariant coordinates of the first adiabatic invariant $\mu$, the magnetic moment; the second adiabatic invariant $K$, related to the bounce period; and $L^{*}$ is the Roederer L-shell parameter.

The RBSPICE SOC lead started the development of a model independent approach to packaging the particle data which would easily be used by the magnetospheric modeling community as a direct input for whatever specific field model desired which could then also provide comparative corrective feedback to the simulation. The work itself started as part of the calibration work between the RBSPICE/TOFxPH data and the ECT/HOPE data with the intent of using coupled energy spectra and PSD plots to better understand the dependencies in the $R_{HR} $ factor (see Sect. 3 – Science Coordination Activities subsection on ECT and RBSPICE cross calibration).

The algorithm currently developed uses the in-situ measured magnetic field in the spacecraft coordinate system i.e., the EMFISIS Level 2 UVW data product (also used for RBSPICE pitch and phase angle calculations). The magnetic moment, $\mu $, is calculated for each energy bin ($E_{i} $) and pitch angle ($\alpha _{j} $) combination for each spin record of the RBSPICE Level 3 PAP data product:

$$ \mu _{i,j} = \left ( \frac{E_{i}}{B_{mag}} \right ) \sin ^{2} \alpha _{j} = \frac{W_{\bot i,j}}{\left \vert B \right \vert}. $$

**Fig. 16:**
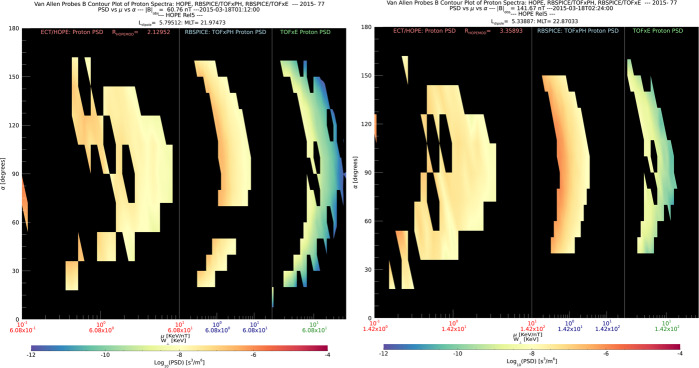
Plot of Phase Space Density (PSD) for two different times using ECT/HOPE, RBSPICE/TOFxPH, and RBSPICE/TOFxE proton data from Spacecraft B during the St. Patrick’s Day Storm in 2015

The observed flux is converted into phase space density using the above equation for energy space integration resulting in a measured value of the distribution function for each value of $\mu _{i,j}$, i.e. $f_{i,j} ( \mu _{i,j}, \alpha _{j}; \vec{r} )$. The magnetic moment is then divided into logarithmic bins, $\mu _{i,j} \rightarrow \mu _{k}$. The PSD values are then binned and normalized into a two-dimensional data structure organized by $\mu _{k}$ and $\alpha _{j}$. Figure 16 shows an example of the calculated PSD data comprised of ECT/HOPE, RBSPICE/TOFxPH, and RBSPICE/TOFxE proton data. Each are plotted into individual spectrogram panels showing logarithmically binned in $\mu $ and organized by pitch angle, $\alpha $. The ECT/HOPE and RBSPICE/TOFxPH proton data overlap in $\mu $ space with RBSPICE/TOFxE proton data at higher values of $\mu $. The figure displays two sets of panels of PSD at different times during the St. Patrick’s Day storm on March 18, 2015 for Spacecraft B. The left three panels reflect a time when the magnetic field is weaker sampling slightly higher values of $\mu $ space compared to the right three panels. The $x$-axis in $\mu $ space and the contour color scale are the same between each set of panels although the although the associated energy range ($\mu (E,\alpha )$ is higher for the earlier panels as is reflected by the secondary $x$-axis displayed for each contour panel. This example calculation displays the capability of generating a combined PSD from ECT/HOPE and RBSPICE data that can be a direct input into modeling systems.

## Science Coordination Activities

One of the key elements of the Van Allen Probes Mission was the intentional attempt to have the instrument teams coordinate science activities both within the mission specific group of instrument teams but also to include external teams such as the team from the Balloon Array for Radiation-belt Relativistic Electron Loss (BARREL) Mission and to also include other assets such as ground radar stations. The most important coordination activities between the instrument teams involved the cross calibration of similar instruments e.g., overlap of proton energy channels between ECT-HOPE and RBSPICE/TOFxPH. The following section describes some of the key coordination activities and results that have been accomplished to this point during the Van Allen Probes Mission.

### Electric and Magnetic Field Instrument Suite and Integrated Science (EMFISIS)

Over the course of the Van Allen Probes mission, EMFISIS conducted various science coordination activities. First and foremost, because magnetometer data is essential for calculating particle pitch angles and field-aligned coordinates for fields data, EMFISIS coordinate with all teams to provide good accuracy magnetic field data in spacecraft coordinates.

Other coordination efforts included:

- Working with the BARREL balloon team to coordinate bust mode data taking at times when the Van Allen Probes spacecraft were magnetically conjugate to regions in which the BARREL balloons were flying. This is described in full detail in the EFW section which follows.
- Coordinating with lightning research ins the US and Hungary to take burst data when over regions where they had good ground measurements and the Van Allen probes were magnetically conjugate to those regions This enabled more detailed studies of lightning-generated whistlers.
- Coordinating with researchers at Goddard Space Flight Center to take burst mode data when the Van Allen probes were at perigee in regions where spread-F is observed. This resulted in some highly detailed observation of spread-F including some unusual observations of a magnetic signatures associated with these waves.
- EMFISIS coordinated efforts to identify times when the Van Allen probes and the Japanese Arase satellite had conjunctions in order to take burst mode data for cross comparisons between the two missions. This has led to several papers on conjugate observations.

In addition to these efforts. EMFISIS did its best to take burst mode data or implement different modes of operation on requests for short periods of time.

### Electric Field and Waves Suite (EFW)

During the Van Allen Probes mission the Electric Fields and Waves (EFW) instrument took part in several collaborative science campaigns with other missions including BARREL, FIREBIRD/Ac6, and WWLLN. These collaborations were focused efforts to collect high time resolution burst waveform data, generally during times of magnetic or drift shell conjunctions.

The three most significant collaborations were:

- BARREL (Balloon Array for Radiation Belt Relativistic Electron Losses) – The EFW and BARREL teams worked closely together for six balloon campaigns in order to determine the temporal and spatial characteristics of magnetospheric waves and resulting electron loss. These campaigns included the 2013 and 2014 Antarctica campaigns (roughly Jan–Feb, 2013 and Dec, 2013–Feb, 2014), three Kiruna, Sweden *turnaround* campaigns (7 balloons in Aug, 2015; 7 balloons in Aug, 2016, 2 balloons in June, 2018), and an Antarctica *superpressure* campaign where a single balloon remained aloft from Dec, 2018 to Feb, 2019. Details on these campaigns from the BARREL perspective are discussed by Woodger et al. (2015), Millan et al. (in prep) and Johnson et al. (2020).
- FIREBIRD (Focused Investigations of Relativistic Electron Burst Intensity, Range, and Dynamics) and AC6 (AeroCube 6) – EFW provided burst 1 collection during times of close magnetic conjunction in order to further understand the connection of magnetospheric waves (primarily chorus) and microburst precipitation. This included several month-long campaigns from 2015–2019 (see Johnson et al. 2020 for details).
- WWLLN (Worldwide Lightning Location Network) – EFW provided burst collection during times when the Van Allen Probes mapped to magnetic field lines over the continental United States in order to study the manner in which lightning activity couples into whistler mode radiation in the inner magnetosphere. The decision to telemeter burst data was based on whether or not significant lightning activity was detected (Zheng et al. 2016).

By mission’s end, EFW had telemetered a substantial dataset of spatially separated, high time resolution data during dynamic times, leading to a number of publications (see Breneman et al. 2022).

#### EFW Campaign with Balloon Array for Radiation-Belt Relativistic Electron Losses (BARREL)

EFW’s first significant collaborative effort was with the Balloon Array for Relativistic Radiation Belt Losses (BARREL) mission of opportunity’s first mission in 2013 (Millan et al. in prep; Woodger et al. 2015). During this roughly two-month long effort the BARREL team launched a total of 20 balloons from SANAE and Halley Bay stations in Antarctica. Balloons had an average duration aloft of approximately 12 days, and typically 6 balloons were aloft at any given time (Woodger et al. 2015). At altitudes of $\sim30\text{--}40~\text{km}$ the balloons measured Bremsstrahlung X-rays created from external sources including electron precipitation from the radiation belts in addition to galactic cosmic rays, solar flares, solar energetic protons. Using a forward folding technique the X-ray spectrum could be reliably used to estimate the spectrum of the incoming flux, particularly when constrained by in situ flux measurements from satellites (see Millan et al. in prep; Woodger et al. 2015 for more details). These measurements filled a gap in the near-equatorial Van Allen Probes observations by allowing a direct measurement of precipitating flux – not typically possible for near-equatorial satellites which cannot resolve the small ($\sim1\text{--}2~\text{deg}$) loss cone.

**Fig. 17:**
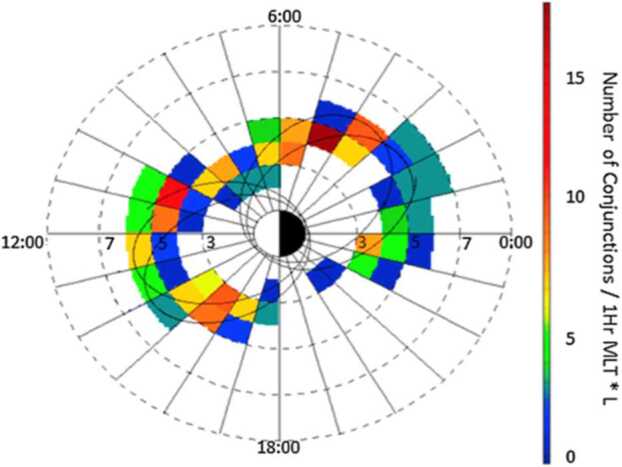
Woodger15 all BARREL/VAP conjunctions 2013–2014

One of the key science goals of BARREL was to quantitatively investigate wave-particle interactions leading electron precipitation by various wave types and other precipitation drivers at times of magnetic or drift shell conjunction. In 2013(2014) conjunctions were focused in the morning(afternoon) sector, as shown in Fig. 17 (derived from Fig. 1 in Woodger et al. 2015). This location played an important role in the EFW burst 1 operation, with morning sector conjunctions typically sampled at the highest rate (16K) in order to resolve chorus waves, and with lower rates for the afternoon sector to resolve lower frequency hiss and EMIC waves.

The EFW/BARREL collaboration was highly successful for both missions; planning and communication between the teams was a key component to this success. An approximate three-day lead time was needed to decide on when burst data were to be collected to ensure the commands would be successful uplinked to the satellites. Shorter timeframes were sometimes available for us to make decisions, however we tried to stick to making decisions about burst collection 3 days out. Thus, it was clear that we would need a clear method to plan and prioritize collection periods.

The relevant teams met informally at AGU a year prior to the first BARREL campaign to discuss strategy. A plan was developed for the BARREL team to create expected trajectories as Google Earth KML files to enable prediction of conjunctions between the balloons and satellites. These plots, which included the balloon flight paths and the magnetic footprint of the Van Allen Probes, ground stations, and other satellites, were then referenced when prioritizing burst collection and download.

Starting in mid-December 2012, when the team declared flight-ready in Antarctica, the BARREL team started sending daily emails that included a high-level summary of the space weather and any potential upcoming activity, followed by updates about which balloons were likely to be launched or terminated, as well as which were still afloat. A list of observed precipitation events followed, along with the current burst data collection times and data in the que to be downloaded. These two pieces of information facilitated discussions between the teams to prioritize downloading data which was likely to be highly impactful. The emails continued with updates from other instruments, missions, and ground observations, along with a more detailed look at the current space environment and predictions of upcoming activity. As these emails were long, they were often ended with a fun fact. This may seem unnecessary to mention or add, but it aided in keeping spirits light which helped with a near 24/7 cadence over a few months.

After the daily emails were sent, the BARREL, EFW, and other instrument and mission teams held a daily phone call to tag up and plan for new burst data collection and downloads. Because we made sure that the emails described above were sent about 2 hours prior to the phone call, our chats were very focused and short. Even with waiting a few minutes at the beginning to make sure everyone was on, say hi, how’s the weather, etc. the average length of time for these telecons was 6 minutes. Telecons were cancelled when not needed and the team worked hard to avoid weekend tag ups to give people some much deserved and needed down time.

As many researchers were interested in the ongoings of the BARREL campaigns but did not want to receive daily emails, we offered a few other forms of communication. The emails were paired down to remove the identification of event times and other potentially sensitive information and then posted to a blog http://relativisticballoons.blogspot.com/. In the later campaigns this blog was used for public outreach and we added a second science focused blog for researchers. We also started posting when we were launching and terminating balloons, along with some other fun information to a twitter account @keV_Balloons, and on to a Facebook page. These interactions provided unexpected engagement with the broader research community. Specifically, the Twitter interactions with other space physics researchers led to the collection of extra ground data and resulted in successful proposals to get time on EISCAT (which was near conjugate to the Kiruna launch site) for the 3rd and 4th BARREL campaigns.

Through advance planning, respecting people’s time, and accommodating their preferred communication format, we were able to have a successful first campaign. This success led everyone to work extra hard, and even look forward to a second and additional intense follow-on campaigns.

Another aspect which enabled the success of BARREL as a mission of opportunity was the openness of the Van Allen Probes Team to include BARREL in other activities. The BARREL team regularly had joint meetings with the EFW and other teams and were always included in the twice-yearly mission meetings. Perhaps most importantly, The BARREL team was included in their efforts for outreach to the broader scientific community. This included the Van Allen Probes Data/Analysis help sessions during posters at AGU and GEM as well as inclusion within chapters such as this one. This was further enabled by the BARREL team ensuring their data was available through CDAWEB and analysis software provided through SPEDAS (Angelopoulos et al. 2019).

#### EFW Campaign with JAXA Arase Mission

The Van Allen Probes EFW instrument team and the Japanese Aerospace Exploration Agency (JAXA) Exploration of energization and Radiation in Geospace (ERG) (Miyoshi et al. 2018 – also referenced as “Arase”) science team collaborated in a prolonged science campaign from 2017–2019 focusing on science topics that motivated the collection of high-rate burst waveform electric and magnetic field data (see Breneman et al. 2022 for more details). This dataset provides simultaneous observations of plasma waves with a range of spatial separations in L and MLT, as well as the first significant dataset of wave observations separated primarily by magnetic latitude during more than 500 magnetic conjunctions. The latter is critical for understanding the way in which plasma waves propagate away from their (typically) near-equatorial source to higher latitudes. Results have led to significant increases in our understanding of the spatial and temporal dynamics of wave/particle interactions in the inner magnetosphere.

**Fig. 18:**
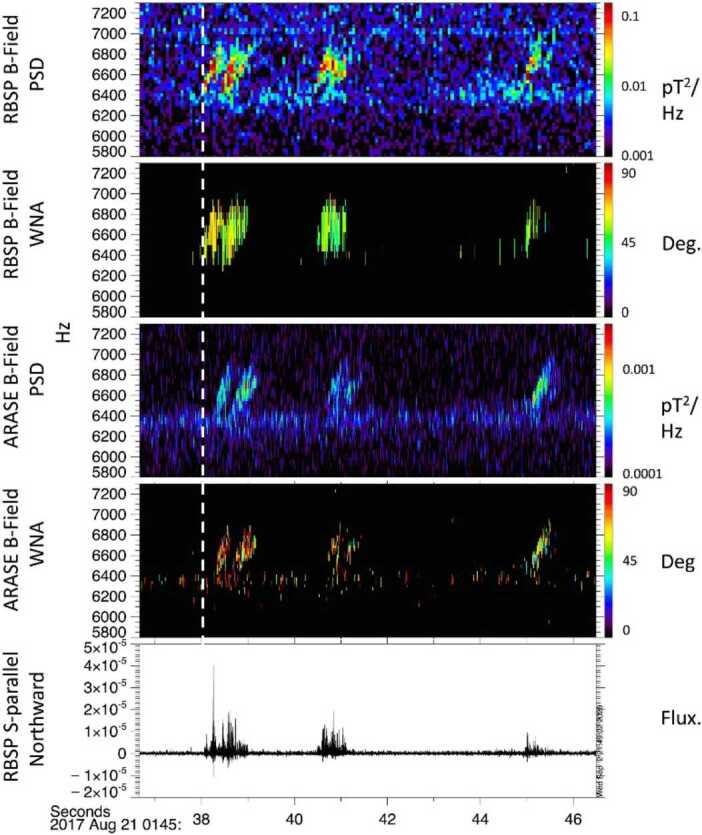
Observations of the RBSP-B and ARASE magnetic field instruments while the spacecraft are on the same magnetic field line and are separated by $\sim20~\text{deg}$ in magnetic latitude. Figure taken from Colpitts et al. (2020)

For example, Colpitts et al. (2020) used simultaneous conjunction observations at magnetic latitudes of 11 deg (Van Allen Probe A) and 21 deg (Arase) to directly show how rising tone chorus packets propagated from their equatorial source to higher magnetic latitudes. Details of this propagation (e.g., ducted vs non-ducted) are critical for determining how chorus waves interact with electrons with energies from tens to hundreds of keV. Figure 18 displays the conjugate observations of the RBSP B Magnetic Field wave measurements on the same time scale as the Arase Magnetic Field wave measurements. It is clear that both spacecraft observe the waves even though they are at different inclinations ($10^{\circ}$ for RBSP and $32^{\circ}$ for Arase (JAXA ARASE Website)).

**Fig. 19:**
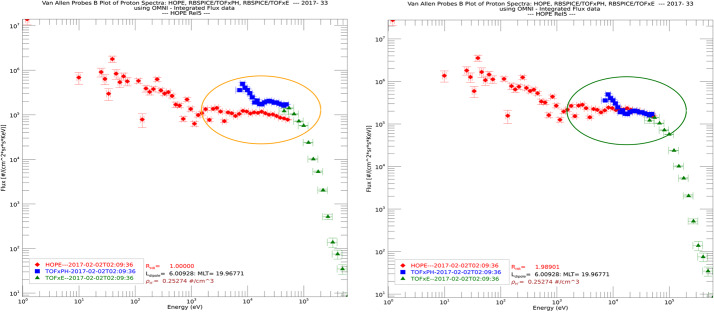
Comparison of ECT/HOPE, RBSPICE/TOFxPH, and RBSPICE/TOFxE spectra at 2017-02-02T02:09:36 UTC using the OMNI data variables from each data product. The left panel shows the raw spectra from each instruments data product with HOPE in red, TOFxPH in blue, and TOFxE in green. There is a clear discrepancy between the RBSPICE TOFxPH/TOFxE OMNI differential flux and that of HOPE as shown by the orange oval. The right panel shows the same data except that the HOPE data has been increased by a factor of $R_{HR} \cong 1.98$ referenced in the figure as the HOPEMOD factor which is used to shift the measurements such that they now form a continuous spectra excluding the lowest TOFxPH energy channels as shown by the green oval. The black circled TOFxPH energy channels lifted above the merger of the HOPE and TOFxPH/TOFxE spectra are due to lower energy oxygen ions in the TOFxPH system being interpreted as protons. The specific factor, $R_{HR}$, used was calculated using a simple algorithm as described in this section

The relative time of arrival difference and wave normal angles for chorus packets identified on both spacecraft were used to constrain a ray-tracing analysis which indicated that the waves were generated near the magnetic equator with nearly parallel wave normal angles and then propagated without any significant density ducting to higher latitudes.

A similar result was provided by Matsuda et al. (2021) for simultaneous observations of EMIC waves on Arase, RBSP, and ground magnetometers and their effect on ion heating. A comprehensive summary of these results and many others from this collaboration is presented in a submitted paper by Miyoshi et al. (2022).

### ECT HOPE and RBSPICE Cross Calibration Factor – $R_{HR}$

The RBSPICE and ECT teams have worked on cross calibration of the species-specific observations between the ECT/HOPE, ECT/MagEIS, and the RBSPICE instrument observations for similar energy channels. These calibration activities resulted in adjustments to the efficiencies in the calibration table for the RBSPICE instrument with additional work still ongoing. One of the key cross calibration activities has been to resolve an apparent discrepancy between the upper energy channels of the HOPE and the lower energy channels of the RBSPICE proton differential flux measurements. As of the writing of this manuscript there is an approximate factor of 2 difference between the HOPE and RBSPICE proton data for the HOPE release 4 data set. Upon analysis, the problem is significantly more complex than a simple multiplicative factor although there is an expectation that some of this discrepancy will be resolved in the upcoming release 5 dataset.

**Fig. 20:**
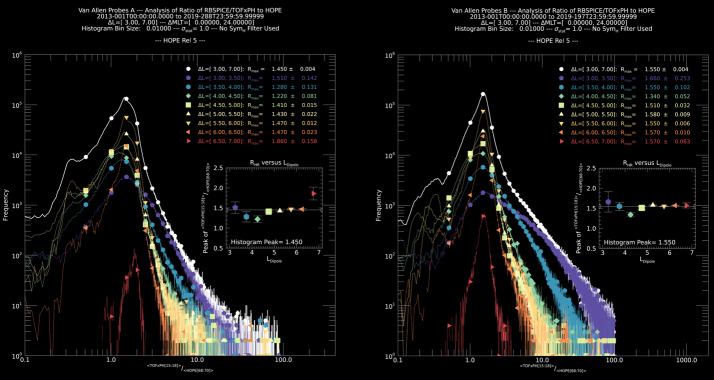
Distribution of the correction factor, $R_{HR}$, for each spacecraft (A-left, B-right) accumulated over the entire mission. Each black curve includes all data and the rest of the curves provide the breakout by ${L}_{Dipole}$ segments between 3.0 and 7.0 in $0.5R_{E}$ increments. The consistency across ${L}_{Dipole}$ is reflective of the significant work to cross calibrate the ECT-HOPE and RBSPICE observations throughout the mission

For example, the left panel of Fig. 20 shows two combined proton spectra using OMNI data from HOPE (red), RBSPICE/TOFxPH (blue), and RBSPICE/TOFxE (green). Error bars reflect the width of each energy channel ($\hat{x}$-axis) and the Poisson counting errors ($\hat{y}$-axis). There is a clear mismatch between the HOPE OMNI differential flux higher energy channel measurements and the RBSPICE/TOFxPH measurements well outside the range of the error bars. In contrast, the TOFxPH and TOFxE measurements form a continuous spectrum within the limitations of the errors.

In the right panel, a simplistic algorithm has been used to match the HOPE upper energy observations with those of the RBSPICE/TOFxPH observations of similar energy. This figure includes a printout line HOPEMOD Factor (t0) which identifies the scalar multiplicative factor used to change the HOPE flux to match that of the RBSPICE/TOFxPH flux for the time 0 observation. In this particular example, the calculation itself is only accurate for the upper energy channels of the HOPE data. This is in part because the lower energy channels of the RBSPICE/TOFxPH data for the observation time is contaminated with accidentals causing the lifting of the TOFxPH spectra (black circled area). For this particular time, the required factor needed to modify the HOPE flux is $\sim1.98$. The algorithm used is described in the following steps:

1. $\langle j_{HOPE} \rangle = \sum _{i=68}^{i=70} E_{i}/{3}$: $\begin{array}[t]{l} E_{range} =[30.3~\text{KeV}\text{--}47.8~\text{KeV}] \Delta E=17.5~\text{KeV}\\ E_{68} =32.7\pm 2.5~\text{KeV}\\ E_{69} =38.1\pm 2.8~\text{KeV}\\ E_{70} =44.4\pm 2.5~\text{KeV} \end{array}$
2. $\langle j_{RBSPIC E_{TOFxPH}} \rangle = \sum _{i=15}^{i=18} E_{i} / {4}$: $\begin{array}[t]{l} E_{range} = [31.2~\text{KeV}\text{--}46.5~\text{KeV}]\Delta E=15.3~\text{KeV}\\ E_{15} =32.9\pm 3.3~\text{KeV}\\ E_{16} =36.3\pm 3.6~\text{KeV}\\ E_{17} =40.1\pm 3.9~\text{KeV}\\ E_{18} =44.3\pm 4.4~\text{KeV} \end{array}$
3. $R_{HR} = \frac{\langle j_{RBSPIC E_{tofxph}} \rangle}{\langle j_{HOPE} \rangle}$
  (Note: $R_{HR}$ is referenced as the HOPEMOD factor, $R_{HOPEMOD}$, or just HOPEMOD in some plots)
4. $j_{HOPE_{ch}} = R_{HR} * j_{HOPE_{ch}}$

This particular algorithm provides a 0th order of calibration between the HOPE and RBSPICE instruments spin-by-spin. There are significantly more complex aspects of this calibration problem that includes positionally where the spacecraft is within the orbit by both L and MLT as well as the ongoing level of magnetospheric activity as Sym-H (or Dst) and whether the spacecraft is within the plasmasphere or outside the plasmasphere.

Figure 20 shows the distribution of the values of $R_{HOPEMOD}$ for the entire mission for both spacecraft (A-left, B-right). In the plots, the black curve displays the distribution for the entire mission for all values of $R_{HOPEMOD}$ within the cutoff limits: $R_{range} = \left [ 0.01, 100.0 \right ]$ and $L_{Dipole} =[3.0 R_{E}, 7.0 E_{E} ]$. The rest of the curves show the distributions of $R_{HOPEMOD}$ of $L_{Dipole}$ between $3.0R_{E}$ and $7.0R_{E}$ in $0.5R_{E}$ increments. Each inset plot displays the location of the peak for each curve with errors calculated based upon the width of the individual peaks.

**Fig. 21:**
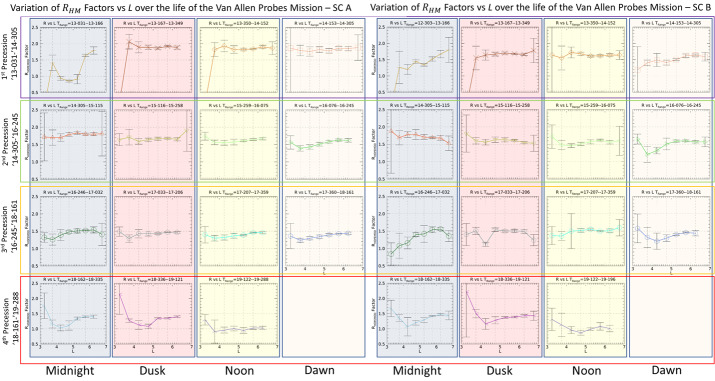
Plot of the $L_{Dipole}$ dependency of $R_{HR}$ for different periods throughout the mission. The dependency on $L_{Dipole}$ is fairly constant throughout the mission except for 1) the initial quarter period (2013-031 through 2013-166) where both instruments are adjusting HV gain to stabilize rates and 2) the final precession period (or so) where the RBSPICE instrument performance has degraded especially for $L<5$

Figure 21 displays these peak measured values for the entire mission for both spacecraft (A-left, B-right) as a function of $L_{Dipole}$ for different times throughout the mission. The time segments each represent one quarter of a precession of the petals of the Van Allen Probes orbits throughout the mission. Each time segment is centered on one of the primary MLT points of Midnight, dusk, noon, or dawn in order of precession periods over the 7-year mission. There are RBSPICE HV gain adjustments in 2013 and 2015 where the ratio of HOPE to RBSPICE/TOFxPH flux observations remains fairly constant for those years but starts to drift downward thereafter. *Error! Reference source not found.* displays the peak measured value of $R$ for different times within the mission (A-top, B-bottom). These curves more clearly show that there is a drift in the $R_{HR}$ value which is indicative of depredation of each of the RBSPICE detectors. Each curve shows a constant value of $R_{HR}$until the final calibration changes in 2015 and thereafter the value degrades. The remaining details of the calibration story of HOPE and RBSPICE proton observations are to be presented in a future paper.

**Fig. 22:**
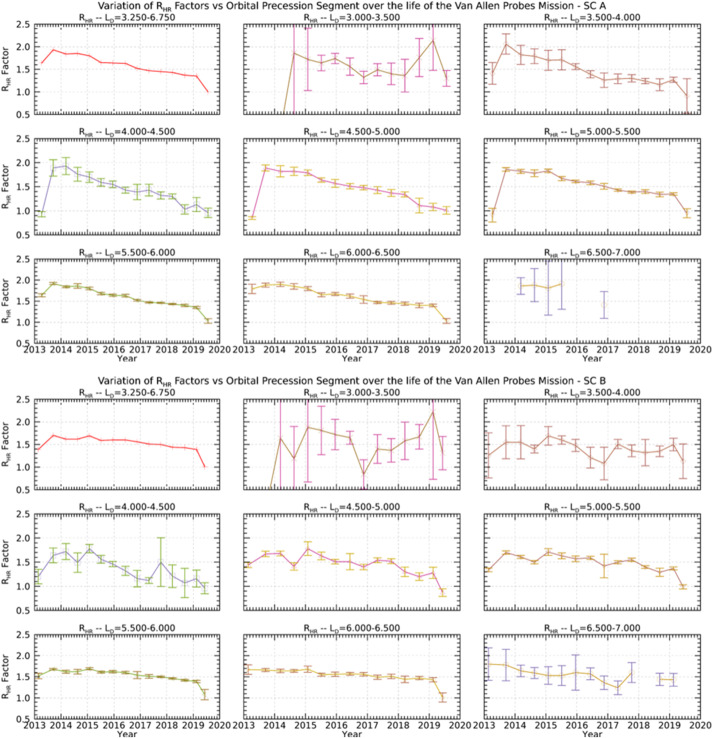
Plot of the $R_{HR}$ for SC-A (top) and SC-B (bottom) for individual segments of $L_{Dipole}$ as a function of Mission time. After the last RBSPICE calibration adjustment in January 2015 there is a slow degradation of the RBSPICE instrument that is captured very clearly in these plots comparing the RBSPICE and HOPE proton flux over time

## Science Analysis Software

Each of the Van Allen Probes Science Operations Centers (SOCs) used existing software or designed and programmed new software to provide a graphical view of the key indicators of instrument performance. The programs used were highly specific to the instruments with fully understanding of instrument variables and parameters allowing the engineering and science teams the capability of fine-tuning instrument performance throughout the mission. The sections below provide details on the software programs used by each of the instrument teams.

### EMFISIS and ECT

While the Electric and Magnetic Field Instrument Suite and Integrated Science (EMFISIS) instrument employed substantial analysis software to generate the L4 WNA products, the software itself was not released to the public. That being said, EMFISIS was one of the primary sponsors of the development of the Autoplot analysis and display tool developed by Jeremy Faden (Faden et al. 2010). This is a free data analysis tool written in Java which allows it run on virtually any OS with Java support. Quoting the Autoplot website (Autoplot.org): “Autoplot is an interactive browser for data on the web; give it a URL or the name of a file on your computer and it tries to create a sensible plot of the contents in the file. Autoplot was developed to allow quick and interactive browsing of data and metadata files that are often encountered on the web. For more information, see Faden et al. (2010) and the introductory PowerPoint slides.”

This tool was originally developed for use by the NASA virtual observatories (VxO’s) but has since been adopted by both the Energetic Particle, Composition, and Thermal Plasma Suite (ECT) and EMFISIS teams as their primary data tool for working with the various measurements made on the Van Allen Probes. Some of the features of the software that were critical to both instrument teams are:

- Reads multiple ASCII formats including Complex ASCII tables; Binary tables; Common Data Format (CDF); NcML; SPASE; Cluster Exchange Format; NetCDF; OpenDAP; HDF5; TSDS; FITS; Excel; Wav; PNG, JPG, etc.
- Data is located with compact URI addresses. These contain the location of the data and additional information needed to use it.
- Special support for CDAWeb server at NASA/Goddard, HAPI, and other data servers.
- Das2 library used to create interactive graphics with slicing and custom interactions.
- Wildcards can be used to aggregate (combine) data from multiple files into one time series.
- Long time series may be rendered as a sequence of images as a “pngwalk” and viewed as a Cover Flow, table of thumbnails, or on a time line.
- Any displayed data may be saved to disk in ASCII, Common Data Format (CDF), and other formats, or plotted as PNG, PDF, or SVG.
- GUI State may be saved as an XML “.vap” file and restored.
- Software may be run client side or server side.
- Data access layer for file reading may be used in MATLAB, IDL, or SciPy (via Java bridge), providing a common interface regardless of data source.
- Scripting via Jython, to control the application and read in data using metadata-aware datasets.
- Open-source (GPL with classpath exception) and may be used

This tool has turned out have enormous value and continues to be used widely by the science community both for Van Allen probes data as well as for other missions.

### Electric Fields and Waves Suite (EFW)

During the lead up to launch, as well as throughout the mission and post mission, the EFW team wrote software as part of the IDL SPEDAS software package (Angelopoulos et al. 2019) intended for data access, calibration, and analysis (currently only bleeding-edge release). See Breneman et al. (2022), for further details.

EFW SPEDAS routines are found in the subfolder /general/missions/rbsp/efw/.

Routines are reliant on other code in the SPEDAS package, spike kernels from the CSPICE package, NASA’s CDF file library, and magnetic field mapping routines in the IDL Geopack package. Installation instructions for these packages can be found at the respective websites.

### Radiation Belt Storm Probes Ion Composition Experiment (RBSPICE)

#### Mission Independent Data Layer – MIDL

The APL SOC/Instrument Team used the MIDL (Mission Independent Data Layer) framework as an important tool to analyze RBSPICE data, develop calibration algorithms and parameters, and validate the official SOC data. MIDL is a code base maintained by APL to analyze and calibrate particles and fields space physics data. MIDL allows the scientist user to read Level 0 and Level 1 data into an analysis framework where it is represented by a mission independent data object type (for example: “Particle Spectral Data”, “Magnetic Field Data”, or “Event Data”). When Level 0 data is read, calibrations are applied to the data to create the particle data object. Attitude and Ephemeris information calculated from the mission SPICE kernels is also applied at this time. The MIDL tool allows customizations of the various calibration parameters (or entirely new calibration files) to be changed interactively.

The MIDL code base contains several interactive tools: spectrograms, one- and two-dimensional histograms, line plots and more, which can be used to visualize the data and test calibration changes. The code base also provided the capability to generate calibrated data that as then written to “quick look” CSV (comma separated value) files. These particular files were critical in validating the official SOC data product files early on in the mission as they provided the capability to independently compare values. The MIDL generated CSV files can also be loaded back into the MIDL software for further exploration without the computational burden of re-applying the calibrations.

Since the MIDL framework is designed to be reusable, it also allows for mission specific code to be added and controlled by the user. For RBSPICE, these additional modules included the following modules:

- $R_{In}$ *vs* $R_{out}$: As part of the RBSPICE calibration process, complicated corrections for saturation and detector dead time had to be developed (aka “$R_{in}$ *vs* $R_{out}$” corrections). The RBSPICE build of the MIDL interactive data analysis application allows the user to change the nine parameters involved “on the fly”. See Level 1 Processing Algorithms in this manuscript for more details as well as Gkioulidou et al. (2022).
- Corrections for “accidental” background events, where time of flight triggers serendipitously coincided with energy background events were developed and tuning parameters can be changed interactively in MIDL.
- The ECT team created a wide range of magnetic ephemeris parameters using different models of Earth’s magnetic field. RBSPICE MIDL allow the user to “attach” any of these 114 parameters to the RBSPICE data.
- RBSPICE summary/browse plots for the full mission were created using MIDL and can be browsed at: http://sd-www.jhuapl.edu/rbspice/data/plots/. The “COMBO” plot, in particular, distills a lot of spectral information into a single image for each day.

APL maintains a website where interested parties can download the MIDL software application and use it to look at RBSPICE data: http://sd-www.jhuapl.edu/rbspice/MIDL/.

## The Van Allen Probes Science Gateway

### Introduction

The primary goal of the Van Allen Probes mission was to “Quantify the processes governing the Earth’s radiation belt and ring current environment as the solar cycle transitions from solar maximum through the declining phase.” The mission consists in a set of several instruments that collects different type of scientific data used to characterize the Earth’s magnetosphere. The Van Allen Probes mission architecture has no centralized Science Operation Center (SOC). Instead, individual instrument suites maintain their own SOCs and serve science data from those SOCs. This approach has the great advantage of leaving the responsibility of processing and delivering the data in the hands of the instrument teams who have the necessary scientific expertise. On the other hand, there is the disadvantage is that the mission lacks of a centralized data center which the scientific community can access all the mission desired data in a single place. To address this shortcoming, the Van Allen Probes mission developed the concept of a “Science Gateway”, which is a web site focused on the science investigation and provides a single point of entry for each instrument SOC. The site, as will be illustrated below, provides access to:

- plot and retrieve scientific data, including Space Weather data
- planning tools, e.g., Multi-Mission Orbit Plotter
- ancillary data, e.g., Ephemerides
- Van Allen Probes related bibliography

The Gateway was developed using “Drupal”, an open-source content management system (http://drupal.org). The usage of Drupal allows registered users to contribute new material and greatly simplifies the maintenance of the site. Although registration is not required to access most of the content of the Gateway, we strongly encourage users to register using the “Create Account” button at the top of the page to take full advantage of all its content.

### Science Gateway Web Interface

**Fig. 23:**
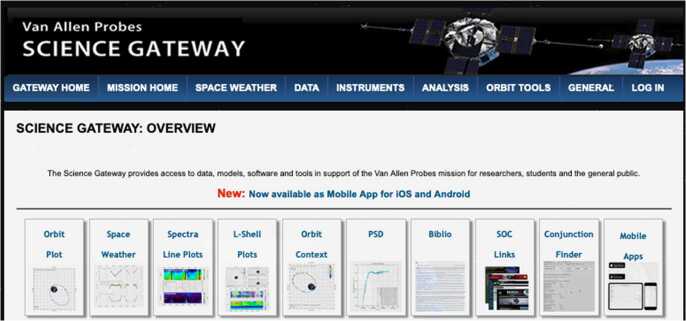
Front page of the Van Allen Probes Science Gateway showing the capabilities available for users

The URL for the Science Gateway is https://rbspgway.jhuapl.edu/ and the front page on the Science Gateway is illustrated in Fig. 23.

The page contains a main menu at the top of the page, and shortcut links for the most used tools in the form of clickable icons/buttons.

### Plotting Utilities

The Van Allen Probes mission contains several instruments, and the needs of the Science Gateway plotting utilities were expected to fit the following requirements:

- Flexible to handle all the data different format from all instruments.
- Able to generate different, high-quality plots (spectrograms, line plots, L-shell, Orbit-context, etc.)
- Capable of handling large amount of data with very little burden on the user/client side.
- Available via web from everywhere, to everyone (on mobile and non-mobile devices).
- Wide range of user customizations.
- Capable of saving the user created plot in the form of a URL, to be retrieved at a later time, and also capable of saving the user plot in PNG or PDF format.
- Allowing users to download the data used to generate the plots in CDF files. These files are not the same as the originally generated SOC files since they contain only a subset of the original data.

The Van Allen Probes Science Gateway can generate plots based on CDF files coming from each instrument SOC. Users have also access to auxiliary data such as DST, Kp indices together with solar wind speed data from ACE/OMNI. Users can also add MLT/MLAT/L-shell as auxiliary x-axis for each spacecraft. Plots are available as spectrograms/line plots, L-shell plots, and orbit-context plots. All links for these types of plots are under the “DATA” in the top main menu. The plotting infrastructure is based on a combination of JavaScript and PHP for the client side, and C-compiled code for the server side.

#### Spectrograms and Line Plots

**Fig. 24:**
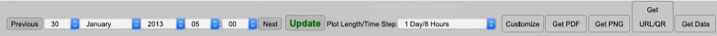
Plotting main user interface for the Van Allen Probes Science Gateway

All Van Allen Probes related data can be used to generate either spectrograms or line plots, depending on the type of data. This includes level 2 (L2), level 3 (L3), level 4 (L4), and Space Weather data. The page main user interface is shown in Fig. 24.

**Fig. 25:**
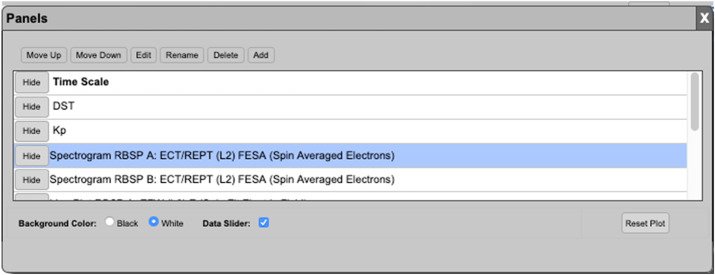
Data selection user interface

Users can select the end of the time interval and its extent back in time. The “Customize” button allows the addition/deletion of plots and also their customization as shown in Fig. 25.

**Fig. 26:**
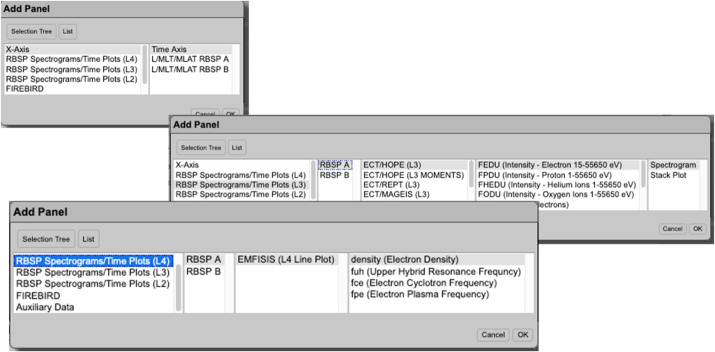
Dialog boxes to create new plots, select input data sets, and data product levels

To create a new plot, a user selects the “Add” button, and the dialog shown in Fig. 26 is displayed.

**Fig. 27:**
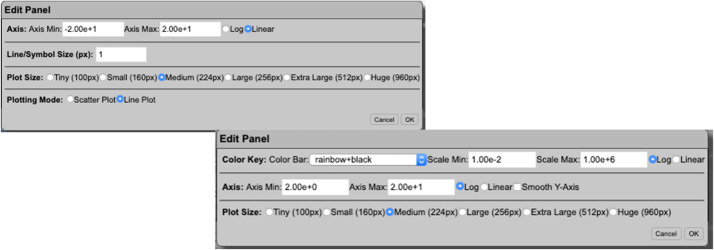
Example dialogue boxes for a Line plot and a Spectrogram

This allows the user to select all Van Allen Probes data, FIREBIRD, and Auxiliary data such as Solar Wind data. Once the data type and plot type (where applicable) are selected and the plot has been generated, it can be further customized by selecting, and clicking the “Edit” button on the main “Customize” dialog. The “Edit” dialog format depends on the type of plot. Figure 27 shows some examples of the dialog boxes displayed for a Line plot and a Spectrogram.

#### Data Slider

When hovering on plots with the mouse, a vertical line will appear as seen in Fig. 30; this is the data slider (as seen in the image on the left) allowing the user to slice the data either horizontally or vertically at a selected time when the user clicks the mouse on the plot. The dialogs on the right of the figure appear.

The Data Slider can easily be disabled by using the “Customize” button.

#### L-shell Plots

**Fig. 28:**
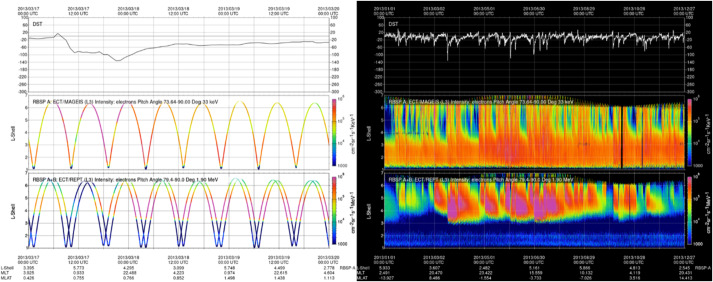
L-shell plot examples for line plots and spectrograms

The Van Allen Probes Science Gateway offers also the capability of creating L-shell plots for spacecraft A, B and their combination as seen in Fig. 28.

Data can be plot in time intervals that range from 6 minutes to 360 days; the data slider is also available for the user to do channel cuts (horizontal) or time cuts (vertical) in the L-shell plots.

#### Orbit Context Plots

**Fig. 29:**
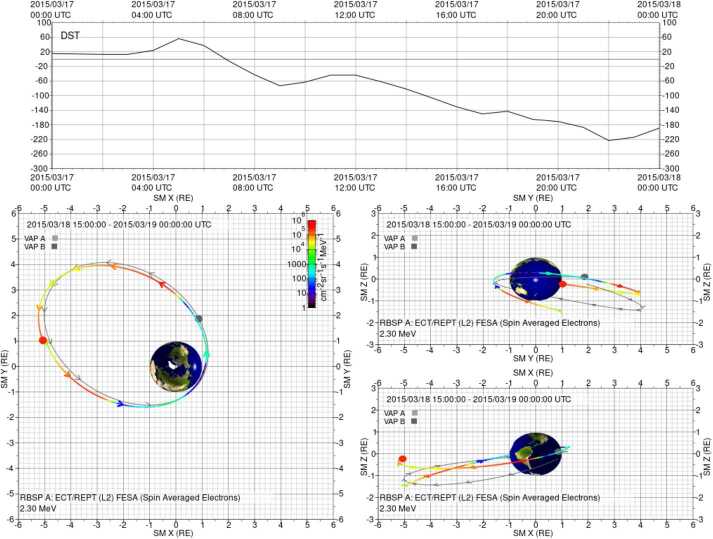
Orbit context plot example showing the value of the Dst index along with the position of the SC in the orbit for the user chosen reference frame

The Science Gateway offers the capability of creating orbit context plots where data from the select spacecraft and instrument are overlaid on the probe orbit at the selected time. See Fig. 29 for an example orbit context plot during the 2015 St. Patrick’s Day storm.

**Fig. 30:**
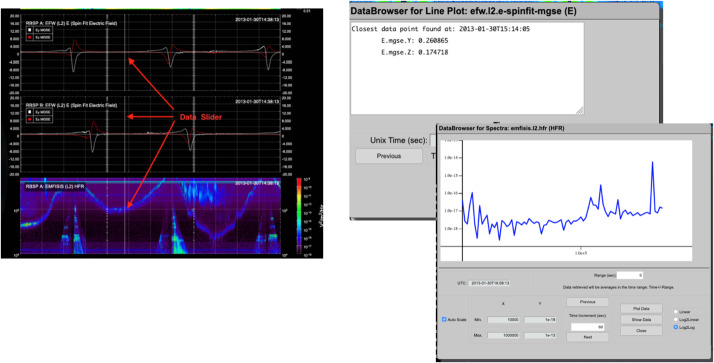
Data slider and associated dialogue boxes

#### Saving Plots

The Plotting infrastructure on the Science Gateway allows 3 different ways to save a plot.

**Fig. 31:**
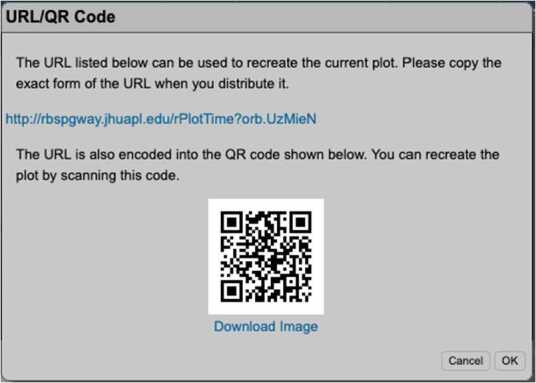
Example QR Code for use to share saved plots and figures with collaborators

1. Downloading the plots as PDF file
2. Downloading the plots as PNG file
3. Generate a unique URL and QR code that can be share with collaborators (see Fig. 31).

### Downloading Plot Data

After creating and customizing plots, users can directly download the data used to generate their plot through an ad-hoc CDF. This file will contain ONLY the mentioned data, and must not be confused with the official instrument SOC generated files. Notice that this feature is available only to users who have an account on the Science Gateway, and have logged into their account. Once the “Get Data” button is clicked, the process of making the ad-hoc CDF file is run on server, and the user will receive an email telling where to download the files and their expiration date.

### Planning Tools

The Science Gateway offers a wide range of web applications that can be used for planning purposes, starting with position calculator, but also orbital tools such as Multi Mission Orbit Plotter and the Conjunction Finder. All these tools will be described below.

#### Multi Mission Orbit Plotter

**Fig. 32:**
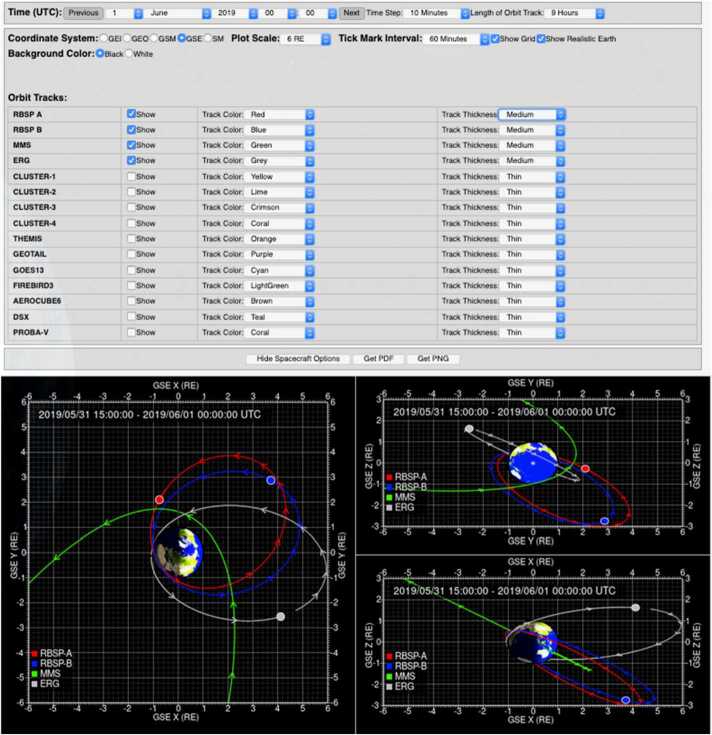
Multi mission orbit plotter example plot showing orbits of the Van Allen Probes A/B, MMS, and ERG spacecraft allowing for visual identification of near conjunctions for coordinated science analysis

This web applications allows to plot orbits for a selected time interval of several space missions, related to the Van Allen Probes. The main interface is illustrated in Fig. 32.

The interface allows to user to customize the plot by

- Selecting the end of the time interval and its extent
- Change the coordinate frame (choices are GEI, GEO, GSM and SM)
- Zoom in or out by changing the “Plot Scale”; units are in $R_{e}$
- The “Tick Mark Interval” allows the user to set when the orbital ticks are to be plotted
- Add or remove a spacecraft, and customize the color used to plot the orbit, and the thickness of the orbital line
- Plots can be downloaded either as PDF or PNG files.

#### Conjunction Finder

This tool allows a user to find times when two selected spacecraft are in conjunction. Unfortunately, the definition of “conjunction” is not unique, and it might entail different conditions for different users. The finder on the Science Gateway uses several user-specified parameters to identify such time intervals when the selected spacecraft are said to be in conjunction. The parameters are

1. $\Delta r$ – Spatial separation between the spacecraft
2. $\Delta \rho $ – Spatial separation between the spacecraft in X–Y plane
3. $\Delta \mathit{mlt}$ – Separation in mlt (magnetic local time)
4. $\Delta \mathit{mlat}$ – Separation in magnetic latitude
5. $\Delta L$ – Separation in L-shell

**Fig. 33:**
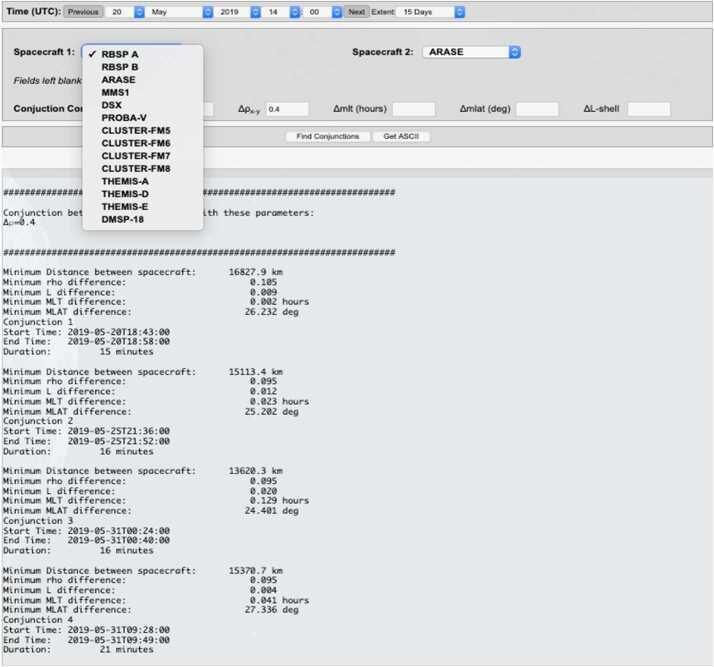
User interface for the conjunction finder tool showing example conjunctions between the RBSP A and ARASE (ERG) spacecraft

Notice that any parameter left blank will not be used. Users can choose to find conjunction between several satellites. The tool is available at https://rbspgway.jhuapl.edu/conjfind and has the interface illustrated in Fig. 33 which shows an example of conjunctions found between the Van Allen Probes SC A (RBSP A) and the ARASE (ERG) spacecraft.

**Fig. 34:**
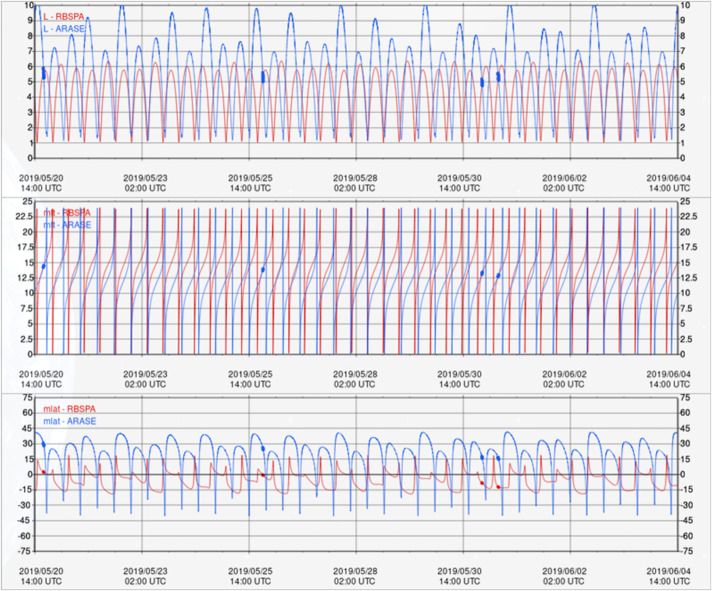
Example set of conjunctions for the RBSP A and ARASE spacecraft shown in an orbit context plot

Furthermore, the tool generates the orbit plots in Fig. 34 to help visualize better the time intervals during which the conjunctions occur. The tool is also useful when working with future orbits of ongoing missions to identify potential conjunctions. As new missions related to Earth magnetospheric and space weather are added to the NASA fleet they will be also added as missions within the conjunction tool providing the ability to work with newer missions than what is currently active.

#### Position Calculator and Orbit Number Calculator

**Fig. 35:**
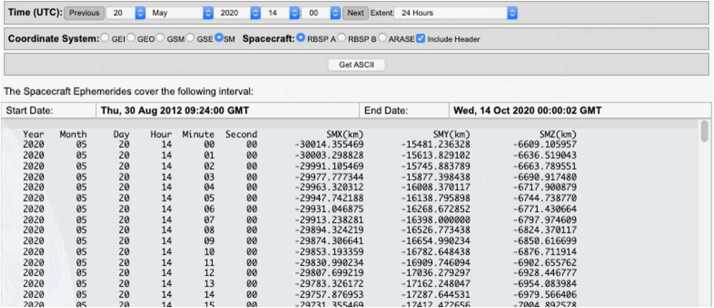
Example generated ephemeris for RBSP A

The Position Calculator generates a spacecraft ephemeris with spacecraft position provided in several reference frames and can be generated for an arbitrary user time interval, see Fig. 35.

**Fig. 36:**
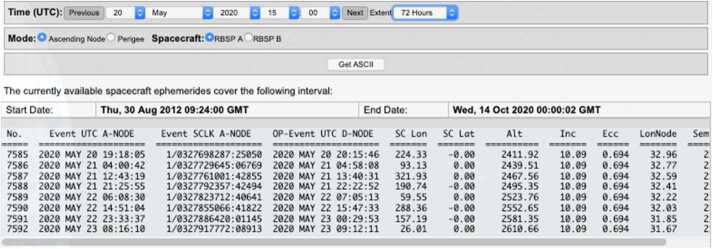
Generation of the orbit number for the RBSP A spacecraft

Similarly, the Orbit Number Calculator generates the orbit number as shown in Fig. 36.

#### Magnetic Footprint

**Fig. 37:**
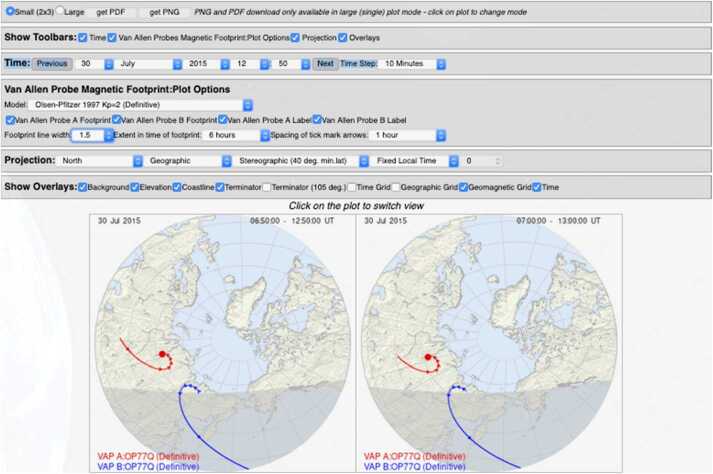
Example plot generated of the magnetic field line footprints for both Van Allen Probes spacecraft as calculated using the Olsen-Pfitzer 1997 Quiet magnetic field model

The magnetic footprint tool allows a user to calculate the magnetic footprint of both Van Allen Probes spacecraft for a selected time interval using ephemerides generated from different magnetic field models such as the Olsen Pfitzer 1997 Quiet (OP77Q) model. The interface is illustrated in Fig. 37. Users can download the customized plots as PNG, PDF or as Goggle Earth KMZ files.

### Van Allen Probes Bibliography

**Fig. 38:**
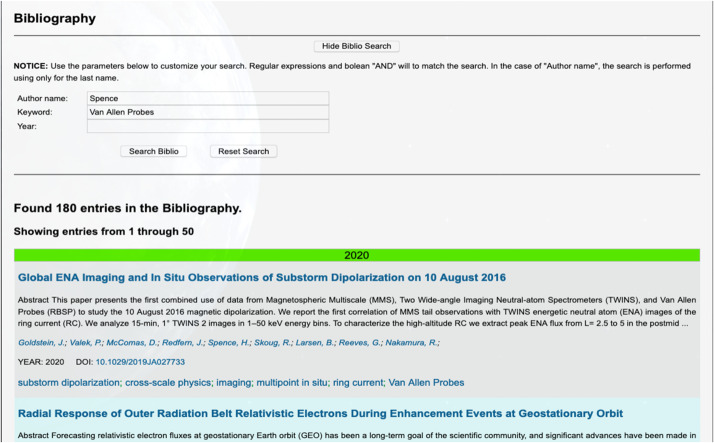
Example search screen from the Gateway Bibliography search page looking for articles written by the author with the last name of “Spence” as a reference to Prof. Harlan Spence the PI of the Van Allen Probes EFW Instrument

The Science Gateway offers the capability of accessing a searchable bibliography of all published manuscripts related to the Van Allen Probes scientific mission and its findings. The bibliography contains currently more than 900 entries, and it is updated on a monthly basis. As illustrated in Fig. 38, the Bibliography user interface offers the capability of searching the bibliographic archive using

- Author Last Name
- Keyword
- Publication Year

**Fig. 39:**
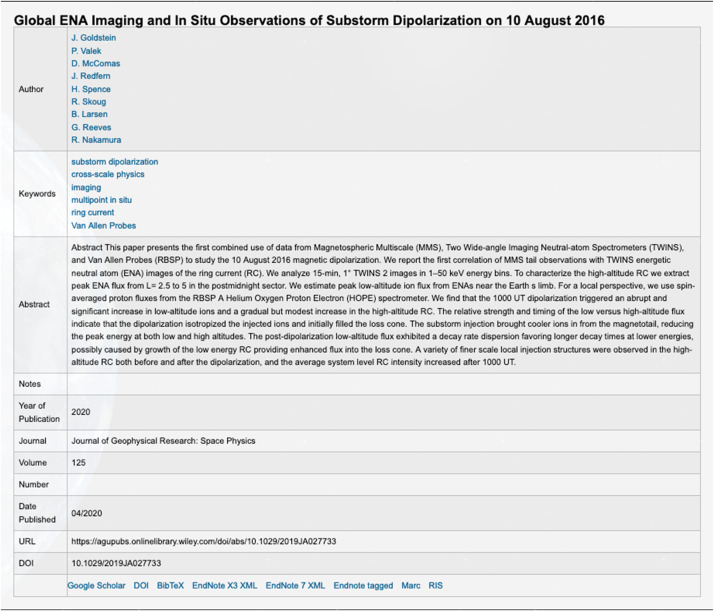
Example returned bibliography entry showing the detail provided with each entry

Each entry contains a link in the publication title that leads to a page that reports the amount of information as illustrated in Fig. 39.

### The TS07D Empirical Magnetic Field Model

The Van Allen Probes Science Gateway serves as the host of the TS07D empirical magnetic field model (Tsyganenko and Sitnov 2007; Sitnov et al. 2008). Empirical magnetic field models have long been useful tools in magnetospheric physics as they allow for determination of the global 3D magnetic field structure. This capability enables observations to be correlated via the technic of field line tracing, a necessary capability in 1) mapping field lines from ground and ionospheric signatures to the magnetosphere and vice versa, 2) determination of spacecraft magnetic conjunctions, and 3) evaluating spacecraft foot points. Additionally, knowledge of the 3D magnetic field is necessary in computing particle adiabatic invariants and tracing particle paths through the magnetosphere.

The general approach to empirical modeling is to formulate an analytical description of the system and then fit the corresponding non-linear parameters and linear amplitude coefficients to the available data. However, this is not straight forward for the magnetosphere. Not only does the magnetosphere react to changes in the solar wind, such as increasing and decreasing in size with changes in solar wind dynamic pressure $P_{Dyn}$, it also undergoes global internal reconfigurations, for example during geomagnetic storms. Furthermore, there is a limited amount of observational data and when considering that the overall volume of the magnetosphere is on the order $\sim 10^{4}R_{E}^{3}$ (limiting the modeling domain to $25R_{E}$ down tail) and at any given moment there is on the order of ten or less magnetospheric spacecraft equipped with scientific magnetometers taking observations above Low Earth Orbit (LEO). The earlier approaches (Tsyganenko 2013 and references therein) were to individually formulate a description of the magnetic field for each of the primary electric current systems: the field-aligned current (FAC), symmetric ring current (SRC), partial ring current (PRC), tail current, and their associated magnetopause currents. The size and magnitude of these systems were made to be predefined functions of solar wind values and geomagnetic indices. The parameters of these functional forms were then found by performing a least-squares regression against the database of the available magnetometer data. The primary shortcoming of this earlier approach is that it is rigid both spatially and temporally.

As an alternative, the TS07D model sought to enable the data to dictate the current sheet morphology (instead of the model developer) using two conceptual advancements. Firstly, the rigid equatorial current descriptions (SRC, PRC, and tail current) are replaced by a single regular expansion with no predefined azimuthal or radial structure (Tsyganenko and Sitnov 2007). Secondly, the dynamical evolution of the system is driven by a simple albeit powerful data-mining technique termed nearest-neighbors (Cover and Hart 1967). The general idea is that during a geomagnetic storm the state of the magnetosphere can be characterized by a finite dimensional State-Space (Vassiliadis 2006). This state-space is constructed from a set of macroscopic parameters derived from solar wind measurements and geomagnetic indices. Temporal progression of geomagnetic storms is then traced through similar trajectories in this State-Space. This provides the mechanism to utilize historical spacecraft observations of the magnetosphere in presumably similar State-Spaces to generate a magnetospheric configuration for the given “observation” time. This bin of similar State-Space data points is then used to fit the model’s non-linear parameters and linear amplitude coefficients. For every subsequent timestep of the model there is then a unique bin of data points used to generate the parameters and coefficients for that unique point in time. The TS07D model is generated utilizing a Five (5) minute time cadence for the generation of the parameters and coefficients providing for a very high time resolution magnetospheric model that can be used in scientific analysis.

Because the magnetometer data now drives the equatorial current structure, the TS07D model is a powerful scientific tool in its own right. The model has been used to contrast the morphology of the ring current dynamics during geomagnetic storms driven by coronal mass injections (CMEs) versus those driven by corotating interaction regions (CIRs) (Sitnov et al. 2008, 2010). A key result of these studies is that during CME driven storms the magnetosphere responds by forming a hook like PRC that closes through a Region-2 FAC. In contrast, during CIR driven storms the formation of the region-2 FAC is inhibited, forcing the PRC to instead close through the magnetopause, resembling a strong tail-like current system. Additionally, the TS07 model has been applied to steady magnetospheric convection events (SMCs) finding the development of two distinctive tail configurations (Stephens et al. 2013).

**Fig. 40:**
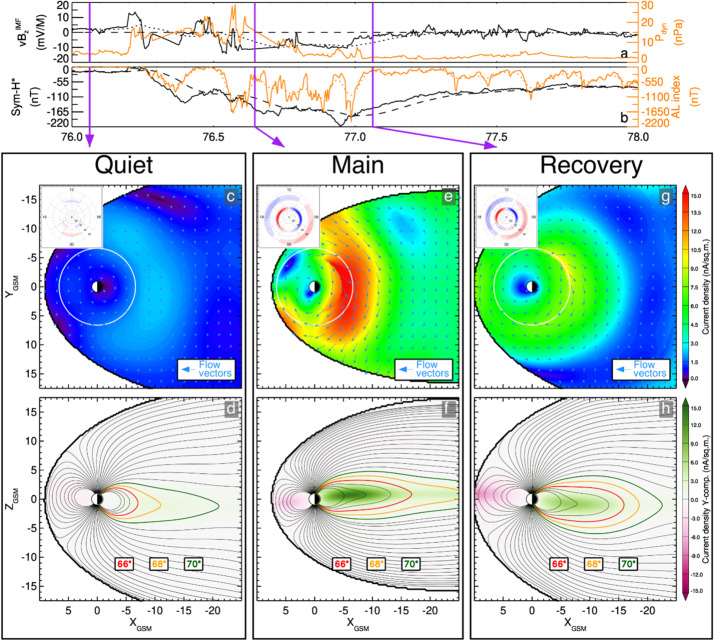
The TS07D reconstruction of magnetospheric current systems during the March 2015 Saint Patrick’s Day geomagnetic storm. (**a**) The solar wind electric field parameter $v B_{z}^{IMF}$ (black line) and dynamic pressure (orange line). (**b**) The geomagnetic indices: pressure corrected storm index Sym-H* (black line) and substorm index AL (orange line). The dashed and dotted lines indicate the smoothed values. The purple vertical bars show the 3 moments of interest, corresponding to (**c** and **d**) the quiet time prior to the start of the storm, (**e** and **f**) the main phase of the storm, and (**g** and **h**) the early recovery phase of the storm. (**c**, **e**, and **g**) Equatorial slices (with no dipole tilt deformation effects) of the current density with the color representing the magnitude and the arrows showing the direction of the current density field. Inset in the upper left of this panel is the current density showing FACs flowing into (blue) and out of (red) the ionosphere. (**d**, **f**, and **h**) The meridional slices of the Y-component of the current density with the color indicating current flowing out of (green) and into (purple) the page. Magnetic field lines are overplotted in black starting from a magnetic latitude of $60^{\circ}$ with a $2^{\circ}$ step size, with three of the field lines being highlighted

Figure 40 showcases TS07D’s reconstruction of the magnetospheric current systems during the March 2015 Saint Patrick’s Day storm, the strongest storm to occur during the Van Allen Probes mission. The left set of panels are of the quiet-time magnetosphere several hours before the CME arrives. All current systems are relatively weak or nearly non-existent, with the only discernable feature being a tail-like current at $7R_{E} \leq r\leq 15R_{E}$. The center panels display conditions during the main phase of the storm, when the storm time index, Sym-H*, reached 150 nT. The whole magnetosphere has become quite compressed as $P_{Dyn} \approx 15~\text{nPa}$. There is a large degree of day-night asymmetry, with equatorial currents generally being much stronger on the nightside. The divergence of the equatorial arrows on the night side at $r\approx 4 R_{E}$ and strength of the Region-2 FACs indicates the formation of a PRC. The nightside field lines are extremely stretched while the dayside is highly compressed. A relatively small amount of the nightside equatorial current flows to noon, with the rest either closing through the ionosphere or outflowing to the magnetopause. The right panels show the magnetospheric conditions ten hours later. By this time solar wind drivers have diminished, allowing the storm to enter the early recovery phase. Although Sym-H* is still similar in magnitude to the center panels, the morphology is quite different. $P_{Dyn}$ has returned to a nominal level allowing the magnetosphere to start expanding back to nominal size. The FAC intensity is similar but they have begun to shift poleward. While a day-night asymmetry still exists in the ring current significantly less of the nightside current closes through the magnetopause and instead closes through a clearly developed SRC. This indicates the particle trajectories are now largely on closed drift paths and that convection of particles on open drift paths has diminished. The nightside field lines are still quite stretched to about $r=15R_{E}$ in which the stretching abruptly stops.

### TS07D Model Architecture

In this section the TS07D model is described in two parts. The first part provides an overview of the model’s architecture including the mathematical description of the magnetic field followed by the nearest-neighbor data-mining algorithm. The subsequent section provides details of the model’s source code and describes where users can find and use the model for their own research.

#### Mathematical Description

Within the magnetosphere, the total magnetic field can be decomposed as the sum of the approximately dipolar internal field and the external field generated by electric currents flowing in space, $\mathbf{B}_{tot} = \mathbf{B}_{int} + \mathbf{B}_{ext}$. Although the internal field is instrumental in determining the magnetosphere’s overall morphology, it originates from the magnetic dynamo deep inside the Earth and is thus beyond the scope of magnetospheric physics. The TS07D model only attempts to capture the external magnetic field and uses the International Geomagnetic Reference Field (IGRF) model (Thébault et al. 2015) to represent the internal field. Although the magnetospheric current systems are interconnected it is useful to model each individually. In the TS07D model the external magnetic field is assumed to be derived from a superposition of the magnetic field generated from equatorial currents, FACs, and magnetopause currents i.e., $\mathbf{B}_{ext} = \mathbf{B}_{eq} + \mathbf{B}_{FAC} + \mathbf{B}_{MP}$.

The first major advancement of the TS07D model was to replace the ad-hoc mathematical descriptions of the SRC, PRC, and tail currents with a single regular expansion (Tsyganenko and Sitnov 2007). This expansion is derived from the general solution of the magnetic scalar potential of a thin current sheet which is used to derive the magnetic vector potential equivalent. The curl of which is expressed in the form:

1 $$ \mathbf{B}_{eq} \left ( \rho ,\phi ,z \right ) = \sum _{n=1}^{N} a_{0n}^{\left ( s \right )} \mathbf{B}_{0n}^{\left ( s \right )} + \sum _{m=1}^{M} \sum _{n=1}^{N} \left ( a_{mn}^{\left ( o \right )} \mathbf{B}_{mn}^{\left ( o \right )} + a_{mn}^{\left ( e \right )} \mathbf{B}_{mn}^{\left ( e \right )} \right ) \# \left ( 1 \right ) $$

where $\mathbf{B}_{0n}^{\left ( s \right )}$, $\mathbf{B}_{mn}^{\left ( o \right )}$, and $\mathbf{B}_{mn}^{\left ( e \right )}$ are the basis fields having symmetric, odd, and even azimuthal symmetry respectively. The basis amplitude coefficients are thus represented by $a_{0n}^{\left ( s \right )}$, $a_{mn}^{\left ( o \right )}$, and $a_{mn}^{\left ( e \right )}$ and are determined when the model is fit to the data. The resolution of the model is determined by the number of expansions represented by $M$ and $N$, corresponding to the azimuthal and radial resolution respectively. If $M$ and $N$ are too small the model will smear out mesoscale features, on the other hand, if they are too large the data will be overfit. The adopted resolution is $\left ( M,N \right ) = \left ( 4,5 \right )$. In order to allow the equatorial current to respond to changes in the solar wind dynamic pressure, $P_{Dyn}$, the coefficients in Eq. (1) are replaced by $a_{\alpha \beta}^{\left ( \gamma \right )} \longrightarrow a_{0,\alpha \beta}^{\left ( \gamma \right )} + a_{1,\alpha \beta}^{\left ( \gamma \right )} \sqrt{P_{Dyn}}$. Panels c, e, and g in Fig. 40 demonstrate how this equatorial description naturally reconstructs tail like currents (Fig. 40c and Fig. 40e), SRCs (Fig. 40g), and PRCs (Fig. 40e and Fig. 40g).

A further complication in the overall modeling is that the Earth’s approximately dipolar magnetic field $\mathbf{B}_{int}$ is not perpendicular to the flow of the solar wind. Near the planet, ($r\lesssim 4 R_{E}$), the magnetosphere morphology generally aligns with the solar magnetic (SM) coordinate system in which the primary axis is the magnetic dipole. Further away ($r\gtrsim 8 R_{E}$), the geocentric solar magnetic (GSM) coordinate system is more appropriate as its primary axis is along the sun-earth line, the approximate direction of the solar wind flow. The angle between these two coordinate systems is termed the dipole tilt angle which continuously changes as the Earth rotates and orbits the sun. The general deformation technique (Stern 1987; Tsyganenko 1998) is employed to account for the dynamics driven by this effect. In particular, this technique is used to bend, warp, and twist the flat current system presented in equation (1) into a shape that more accurately reflects the actual configuration of the equatorial currents (Tsyganenko 2002) due to dipole tilt angle effects. Figure 40d, Fig. 40f, and Fig. 40h demonstrates the impact of the dipole tilt deformation on the equatorial currents. Notice how near the planet the current sheet is perpendicular to the dipole axis but further down the tail it aligns with the Sun-Earth axis instead.

The ionosphere connects to the magnetosphere via the FACs. When observed by low earth orbiting spacecraft, these appear as two sets of concentric ovals (Iijima and Potemra 1976), the higher and lower latitude ovals termed Region-1 and Region-2 FACs respectively. TS07D describes these by bending a model of purely radially directed conical currents sheets $\mathbf{J} = J_{r} \hat{\mathbf{r}}$ (Tsyganenko 1991) to match the realistic shape of magnetic field lines (Tsyganenko 2002) using the general deformation technique, which also accounts for both the day-night asymmetry and dipole tilt effects. Two such systems are used, one for Region-1 FACs and one for Region-2 FACs, with the latter being allowed to rotate in local time. Two free parameters are introduced that allow the systems to independently shift equatorward and poleward. An ionospheric slice of the FACs is inset in Fig. 40c, Fig. 40e, and Fig. 40g, showing their evolution during the March 2015 Saint Patrick’s Day storm. The divergence of the equatorial arrows on the night side, at $r\approx 4 R_{E}$ and $r\approx 6 R_{E}$ for the main and recovery phases respectively, shows how the Region-2 system interacts with the equatorial system to naturally form a PRC.

The TS07D model assumes a closed magnetosphere, that is, the total magnetic field does not penetrate the magnetopause boundary, which can be represented by $\mathbf{B}_{tot} \cdot\mathbf{n} \vert _{S} =0$, where $S$ is the model’s magnetopause surface and $\mathbf{n}$ is the normal on that surface. The only field not yet defined is the field generated by the magnetopause currents, $\mathbf{B}_{MP}$, indicating that the above general constraint is used to derive the currents that define this field. Although in the real magnetosphere $\mathbf{B}_{MP}$ is generated by magnetopause currents, it is convenient to limit the domain of the model to just inside the boundary. The result is that $\mathbf{B}_{MP}$ is curl free and can be represented by a magnetic scalar potential, $\mathbf{B}_{MP} =- \nabla U_{MP}$, the solution of which is found by solving Laplace’s equation $\nabla ^{2} U_{MP} =0$ using separation of variable resulting in a regular expansion form for $U_{MP}$. Each of the magnetic fields inside the magnetopause is given a complementary shielding field such that $\mathbf{B}_{MP} = \mathbf{B}_{int}^{\left ( sh \right )} + \mathbf{B}_{eq}^{\left ( sh \right )} + \mathbf{B}_{FAC}^{\left ( sh \right )}$. The exact form for $U_{MP,int}$, $U_{MP,eq}$, and $U_{MP,FAC}$ depends on the underlying geometry of the field that is being shielded. For example, $U_{MP,int}$ (appendix of Tsyganenko 1998) and $U_{MP,FAC}$ (Eq. (34) of Tsyganenko 1995) use an expansion of Cartesian harmonics while $U_{MP,eq}$ is formulated using Fourier-Bessel harmonics (Eq. (20) of Tsyganenko and Sitnov 2007). The coefficients of these expansions are determined by sampling the model’s magnetopause boundary and minimizing $\left ( \mathbf{B} + \mathbf{B}^{( sh )} \right ) \boldsymbol{\cdot}\mathbf{n}$ using linear least squares regression.

In order to ensure pressure balance, when the solar wind pressure, $P_{Dyn}$, increases the magnetosphere compresses and likewise decreases in $P_{Dyn}$ causes expansion of the magnetosphere. As with nearly all other empirical magnetic field models (Tsyganenko 2013), the TS07D model assumes the entire magnetosphere expands and contracts in a self-similar fashion. This is mathematically represented by a simple rescaling of the position vector $\mathbf{r}' = \mathbf{r} \left ( {P_{Dyn}} / {P_{Dyn,0}} \right )^{\kappa} $, where $P_{Dyn,0} =2~\text{nPa}$ is the baseline dynamic pressure and $\kappa $ is taken to be 0.155 (Shue et al. 1998).

#### Database of Spaceborne Magnetometer Data

The spacecraft in the magnetometer database were chosen to overlap with the advent of continuous solar wind monitoring with the launch of the WIND spacecraft in late 1994 and the ACE spacecraft in 1997. Originally, the TS07D model was constructed with data from the Geotail, Cluster, Polar, GOES 8, 9, 10, and 12, Imp-8 missions (Tsyganenko and Sitnov 2007). The database was later expanded to include the twin Van Allen Probes and the five THEMIS spacecraft (Stephens et al. 2019). This new database also reprocessed and was extended with the Cluster and Polar datasets.

The IGRF model field is subtracted from the spacecraft measurements so that only the external magnetic field remains. The vectors are then averaged to a 5 min cadence when the spacecraft are within $r<5.0 R_{E}$ and 15 min cadence when $r\geq 5.0 R_{E}$ to reflect the slower spacecraft orbital speeds. The data was filtered to limit the radial extent from $1.5 R_{E} \leq r\leq 31 R_{E}$. The lower limit eliminates potentially problematic measurements where the internal approximately dipolar field is relatively large making it difficult to resolve the external field given the limitations of the observations and their associated errors. The upper limit corresponds to the largest apogee from the THEMIS mission. The sparsity of Geotail, THEMIS, and IMP-8 data beyond this distance was found to make the fitting process unstable if included.

In total the database contains 3,589,288 records and is publicly available at the Space Physics Data Facility (CDAWeb) at the following URL: https://spdf.gsfc.nasa.gov/pub/data/aaa_special-purpose-datasets/empirical-magnetic-field-modeling-database-with-TS07D-coefficients/.

#### Data Mining

The second major advancement in the TS07D model is the application of data-mining to determine the dynamical evolution of the model. For any particular moment in time, the Nearest-Neighbor (NN) approach (Cover and Hart 1967; Mitchell 1997; Sitnov et al. 2008, 2012) identifies many other moments when the magnetosphere is in a similar configuration, allowing for a unique bin of magnetometer data used to fit the model which is then repeated for each timestep. In this approach, the magnetosphere is assumed to be characterizable by a finite set of macroscopic parameters which form the components of a time-dependent state vector $\mathbf{G} (t)$ (Vassiliadis 2006) which resides in a State-Space. Since TS07D is a storm-time model the components of $\mathbf{G} (t)$ are formulated from three parameters that generally characterize storms: the solar wind electric field $v B_{z}^{IMF}$, the storm index *Sym-H*, and the time derivate of *Sym-H*.

A major driver of geomagnetic storms is a strong and prolonged southward interplanetary magnetic field (IMF). In particular, the solar wind electric field parameter $v B_{z}^{IMF}$ (defined as the $X_{GSM}$ component of the solar wind bulk flow velocity multiplied by the $Z_{GSM}$ component of the IMF propagated in time to the bow shock nose) is directly related to storm time indices (Burton et al. 1975). As the westward flowing ring current intensifies during a storm, the horizontal (H) component of the magnetic field at mid and low-latitudes decreases as observable by ground-based magnetometers. By averaging across a collection of mid-latitude ground-based magnetometers positioned around the globe a longitudinally symmetric H component index, *Sym-H*, is computed (Iyemori 1990). *Sym-H* can be considered a higher resolution version of the *Dst* index (Wanliss and Showalter 2006). Here, a dynamical pressure correction is applied $\textit{Sym-H*} =A\cdot\textit{Sym-H}-B\cdot \left ( P_{dyn} \right )^{1/2}$ (Gonzalez et al. 1994 and references therein) where $A=0.8$ and $B =13.0$ (Tsyganenko 1996). Furthermore, the values are smoothed by convolving them with cosine windows (Sitnov et al. 2012):

2 $$\begin{aligned} G_{1} \left ( t \right ) &= \left \langle \textit{Sym-H*} \right | \propto \int _{{- \Pi} / {2}}^{0} \textit{Sym-H*} \left ( t+\tau \right ) \cos \left ( {\pi \tau} / {\Pi} \right ) d\tau \# \left ( 2 \right ) \end{aligned}$$

3 $$\begin{aligned} G_{2} \left ( t \right ) &= D \langle \textit{Sym-H*} | / {Dt} \propto \int _{{- \Pi} / {2}}^{0} \textit{Sym-H*} \left ( t+\tau \right ) \cos \left ( {2\pi \tau} / {\Pi} \right ) d\tau \end{aligned}$$

4 $$\begin{aligned} G_{3} \left ( t \right ) &= \langle v B_{z}^{IMF} | \propto \int _{{- \Pi} / {2}}^{0} v B_{z}^{IMF} \left ( t+\tau \right ) \cos \left ( {\pi \tau} / {\Pi} \right ) d\tau \end{aligned}$$

where the operators $\langle \dots | $ indicate that the limits of integration are only over past data. The proportionality signs reflect that the components of $\mathbf{G} (t)$ are normalized to give each dimension of the State-Space similar scale lengths. A half window ${\Pi} / {2} =6$ hours is used to eliminate higher frequency oscillations caused by noise and shorter time scale dynamics such as substorms. The impact of the smoothing process is plotted as the dashed and dotted lines in Fig. 40a and Fig. 40b.

As a storm progresses in time, $\mathbf{G} (t)$ traces a trajectory in the 3D State-Space. For the moment of interest $t '$, there are other storms in which $\mathbf{G} (t)$ are close in the State-Space (for example see Figs. 2 and 3 in Sitnov et al. 2008). Once discretized $\mathbf{G} (t)$ becomes a set of individual points. Now for the moment of interest $t '$ there is a set of $K_{NN}$ other points $\left \{ \mathbf{G} ( t_{i} ) \right \}$which are closest to $\mathbf{G} \left ( t ' \right )$ (its nearest-neighbors or NNs). Here the standard Euclidean metric is used to measure distance. Many of the points in the set of NNs will be adjacent in time as they represent segments of storms in the State-Space. Each collection of adjacent NNs thus has a corresponding time interval associated with it. These time intervals are then intersected with the database of magnetometer data as described above to assemble a unique bin for the moment of interest $t '$, thus mining the database for other data when the magnetosphere was most similar to $t '$. This unique bin of data is then used to fit the model resulting in a unique set parameters and coefficients for that moment. The non-linear parameters are fit using the down-hill simplex method while the linear amplitude coefficients utilize the singular value decomposition method for linear least squares (Press et al. 1992) by minimizing the difference between the modeled and observed magnetic field vectors (Tsyganenko and Sitnov 2007). The number of NNs was chosen to be $K_{NN} =8{,}000$, roughly one NN per $R_{E}^{3}$. This process is repeated for each time step, thus allowing the data to dictate the model’s dynamical evolution.

Throughout this work the source of the data used for the solar wind parameters and geomagnetic indices is the bow shock nose propagated 5 min cadence OMNI database (https://omniweb.gsfc.nasa.gov/ow_min.html), which compiles data from the IMP-8, ACE, WIND, Geotail, and DSCOVR missions as well as the World Data Center for Geomagnetism, Kyoto (http://wdc.kugi.kyoto-u.ac.jp/).

The above procedure for determining a unique bin of magnetometer data and resulting set parameters and coefficients has been performed for each timestep from the beginning of 1995 through the end of 2018 using the same 5 min cadence. The next section details how users can access the source code and run the TS07D model for themselves.

### TS07D Model Usage

The model source code is hosted on the Van Allen Probes Science Gateway under ‘Analysis’ → ‘Model’ → ‘Empirical Geomagnetic Field Models’, or at the following link: https://rbspgway.jhuapl.edu/sites/default/files/SpaceWeather/ts07dmodel_july2017update.for. It is coded using a FORTRAN 77 style syntax which can readily be compiled using the freely available GNU Fortran compiler (https://gcc.gnu.org/fortran/).

The two primary advancements realized in the TS07D model, the regular expansion description of the equatorial current systems and the data-mining driven dynamical evolution, both increase the complexity of the source code as compared to most other Tsyganenko models, which are available on Professor Tsyganenko’s website (http://geo.phys.spbu.ru/~tsyganenko/modeling.html). The former requires a large number of shielding coefficients. Typically, these are hard-coded, but here that is impractical. The later results in a unique set of non-linear parameters and amplitude coefficients for each moment in time. Both of these necessitate additional configuration steps. Users are encouraged to refer to the following example program provided on the gateway: http://rbspgway.jhuapl.edu/sites/default/files/SpaceWeather/ts07d_geopack_example_july2017update.for.

First, users must download the zip file containing static coefficients from the gateway onto their local machines (http://rbspgway.jhuapl.edu/sites/default/files/SpaceWeather/TAIL_PAR.zip) and then the file must be unzipped. Next, the coefficients from the files must be parsed and stored into the common blocks TSS, TSO, and TSE (see the example program). This step must be performed before the model can be evaluated, but it only needs to be done once.

The next step is to load the time dependent inputs. This includes the variable set of parameters and coefficients as well as the solar wind dynamical pressure $P_{Dyn}$. The coefficient files have been generated from the beginning of 1995, corresponding to the beginning of continuous solar wind monitoring by the WIND spacecraft, through the end of 2018 at a 5 min cadence and are located on the gateway at https://rbspgway.jhuapl.edu/new_coeffs_mag_models_v02. They are compressed into tar archives for each day ($\sim210~\text{KB}$), year ($\sim75~\text{MB}$), and the complete set ($\sim1.7~\text{GB}$). Once downloaded, they will need to be decompressed and can then be parsed and loaded into the /PARAM/ common block. The format is human readable ASCII and each entry has been annotated with a brief description. Additionally, the dipole tilt angle and $P_{Dyn}$ are appended to the end of the file and can also be parsed. $P_{Dyn}$ needs loaded into the /INPUT/ common block, while the dipole tilt angle is passed into the subroutine as an argument. Again, all these steps are demonstrated in the sample program. As these inputs are a function of time, every time the user wishes to change the time step, this process must be repeated.

Finally, now that the static shielding coefficients, the variable parameters/coefficients, and $P_{Dyn}$ have been loaded into common blocks, the top-level subroutine *TS07D_JULY_2017* can be called. Note, the signature of this subroutine mirrors that of all the other Tsyganenko magnetic field models, allowing the model to plug into Professor Tsyganenko’s Geopack tracing routines. The subroutine requires six inputs, IOPT, PARMOD, PS, X, Y, and Z. The integer IOPT allows the user to break out the individual field components: $0= \mathbf{B}_{ext}$, $1= \mathbf{B}_{MP}$, $2= \mathbf{B}_{eq}$, $3= \mathbf{B}_{FAC}$. The double array PARMOD is required for consistency with other Tsyganenko models but is not used in the TS07D model and is thus a dummy input. PS is a double representing the dipole tilt angle in radians and is appended to the coefficient files described above. The X, Y, and Z inputs are the Cartesian coordinates representing the GSM position in which the model will be evaluated. The units are in Earth radii using the standard geomagnetism radius of $1R_{E} =6{,}371.2~\text{km}$. Note, the model will return values even when the supplied position is beyond the modeled magnetopause. To determine if the position is within the modeled magnetopause the subroutine *T96_MGNP_D* must be called. The output of the model is BX, BY, and BZ which correspond to the magnetic field in GSM coordinates in units of nT. The complete set of inputs needed to evaluate the model are summarized in Table 6 The top level TS07D subroutine TS07D_JULY_2017 arguments.

**Table 6:** The top level TS07D subroutine TS07D_JULY_2017 arguments

Name	Type	Input/output	Frequency	Description
BXTS, BYTS, BZTS, BXTO, BYTO, BZTO, BXTE, BYTE, BZTE	Double Arrays	Input Common Block	Once	Set of static shielding coefficients for the equatorial currents
A	Double Array	Input Common Block	Every time step	An array containing the parameters and coefficients
PDYN	Double	Input Common Block	Every time step	Solar wind $\boldsymbol{P}_{\boldsymbol{Dyn}}$ (nPa)
IOPT	Integer	Input	Always	Option to switch between the total model and its individual constituents
PARMOD	Double Array	Input	Always	Not used
PS	Double	Input	Always	The dipole tilt angle (rad)
X	Double	Input	Always	Supplied $\boldsymbol{X}_{\boldsymbol{GSM}}$ position ($\boldsymbol{R}_{\boldsymbol{E}}$)
Y	Double	Input	Always	Supplied $\boldsymbol{Y}_{\boldsymbol{GSM}}$ position ($\boldsymbol{R}_{\boldsymbol{E}}$)
Z	Double	Input	Always	Supplied $\boldsymbol{Z}_{\boldsymbol{GSM}}$ position ($\boldsymbol{R}_{\boldsymbol{E}}$)
BX	Double	Output	Always	The modeled $\boldsymbol{B}_{\boldsymbol{x},\boldsymbol{GSM}}$ field (nT)
BY	Double	Output	Always	The modeled $\boldsymbol{B}_{\boldsymbol{y},\boldsymbol{GSM}}$ field (nT)
BZ	Double	Output	Always	The modeled $\boldsymbol{B}_{\boldsymbol{z},\boldsymbol{GSM}}$ field (nT)

In order to evaluate the total magnetic field, the TS07D model must be used alongside a model for the internal magnetic field $\mathbf{B}_{int}$, such as the IGRF model (Thébault et al. 2015), an implementation of which is included in Professor Tsyganenko’s Geopack library which can be found at his website: http://geo.phys.spbu.ru/~tsyganenko/modeling.html. Additionally, several other useful utilities, including geophysical coordinate conversions and magnetic field line tracing are included in the Geopack library.

The TS07D model has also been incorporated into the IDL Geopack DLM (http://ampere.jhuapl.edu/code/idl_geopack.html), making the model available in the Interactive Data Language (IDL) programming language as a dynamic link module (DLM), as well as the International Radiation Belt Environment Modeling (IRBEM) FORTRAN library (https://sourceforge.net/projects/irbem/). The IRBEM library also includes IDL and MATLAB wrappers.

## Lessons Learned

With a mission are large and complex as Van Allen Probes it is bound that there will be numerable opportunities for the instrument teams to find alternative and more efficient methods to handle tasks and processes than what was originally planned. The following section provides details from most of the instrument teams on specific lessons garnered during the over seven-year mission.

### Electric and Magnetic Field Instrument Suite and Integrated Science (EMFISIS)

To a great extent, EMFISIS SOC operations have proceeded as expected and without significant hiccups. However, one key lesson worth noting is the use of Autoplot (described above) for both spacecraft integration and test as well as for flight. While this required early development of software to take spacecraft data packets and put them into the CDF data format that EMFISIS uses for its data products, it paid large dividends in not having to go through a second software development cycle for flight data as is common for many instruments on a variety of NASA missions. An additional benefit was that the EMFISIS team had good experience in looking at the data using Autoplot prior to launch which allowed very quick verification of proper instrument operations early in the mission.

### Electric Field and Waves Suite (EFW) – Lessons Learned

#### Efficiency of Burst 1 Collection

##### List of Constraints

1. sample rate (chorus or EMIC?)
2. How quiet or active things are or are predicted to be
3. Science focus (close conjunctions, loose drift conjunctions?)
4. How much interesting data is currently in memory, and how long will it take to play back?
5. How many spacecraft contacts are available in the next few days?
6. Amount of burst 1 *hopscotch* required.
7. Exhaustion of Tohban,^1^ etc.
8. Prediction of future conjunctions, balloon launches. High altitude winds? Number of simultaneous balloons desired. FIREBIRD campaign focus; WWLLN predictions.

##### Operation of the EFW Burst 1 Instrument

This chapter describes the operation of EFW’s burst 1 waveform memory, a 30 GB solid state (ground-commanded) memory used to store DC-coupled high cadence 3d electric and magnetic field waveforms. This memory was orders of magnitude larger than any previously flown on an EFW instrument and allowed continuous waveform collection for long durations at rates from 512–16,384 samples/sec. Table 7 presents a breakdown of instrument operation and data collection over the entire mission. Varying collection rates from 512 to 16,384 samples/sec were utilized to target interesting waves and structures in different regions of the magnetosphere. Further details of this data product are presented in Breneman et al. (2022).

**Table 7:**
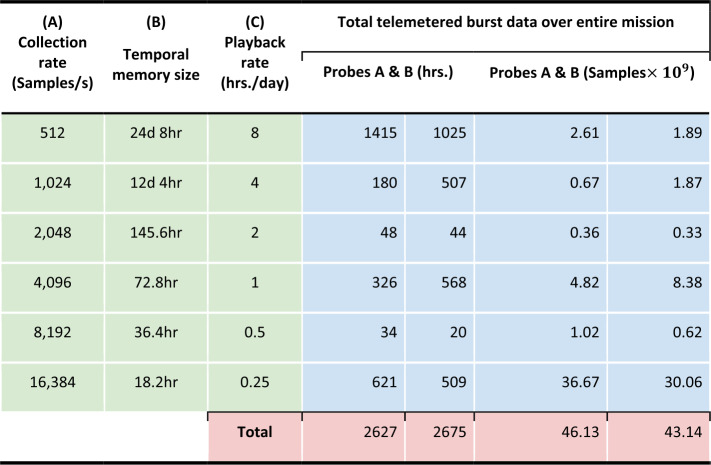
EFW Burst 1 capabilities. The green values show all the possible collection rates that were used during the mission, each corresponding to a maximum number of hours of continuous data collection, and a set number of hours of playback per day. Note that these values assume that the EB1 data is telemetered. The playback rate increased by 30% after stopping to telemeter EB1 in 2013. The blue values show for each Probe and mode the total telemetered data volume (hrs) over the entire mission, and the total number of burst data samples (x109). The totals over all the modes are shown in red

The burst 1 capabilities made EFW well-suited to focus data collection during targeted science opportunities. For example, higher collection rates targeting chorus waves were generally used in the morning sector, while lower rates targeting EMIC waves were generally used in the afternoon sector. The large memory size allowed ample time to evaluate what to telemeter based on inspection of survey data, particularly at lower collection rates.

In addition, burst 1 collection was often focused during times of close conjunctions between the two probes (lapping events), and this data was used to determine the spatial size of chorus and EMIC wave packets (see Breneman et al. 2022). EFW also took part in a number of collaborative campaigns by providing high-rate collection during magnetic conjunctions with other missions (BARREL, FIREBIRD, and WWLLN) mentioned in Sect. 3.2.

By mission’s end, EFW had telemetered a substantial dataset of spatially separated, high time resolution data during dynamic times, leading to a number of publications (see Breneman et al. 2022).

##### EFW Approaches for Increasing Burst Data Return

Unlike more traditional burst memories, burst 1 memory was ground-commanded by an EFW Duty Scientist who decided when to collect, at what rate, and what parts of memory to designate for collection or preservation. Collection was often tailored to a particular science focus such as observations of a particular wave type. For example, collection in the tail region was often tailored to capture VLF waves associated with dynamic injection events. Due to telemetry constraints, typically only a small fraction of collected data was telemetered.

The decision to play back data was based on survey data (e.g. EFW or EMFISIS, or data from other missions), predicted activity levels, or spatial proximity to another payload (e.g. magnetic conjunctions). Examples include: playback during close approaches of the two Van Allen Probes designed to capture a large spread of spacecraft separations needed to determine the scale sizes of chorus waves; playback during the BARREL and FIREBIRD campaigns focused around magnetic conjunctions; and playback during the WWLLN campaigns based on the amount of lightning activity observed.

**Fig. 41:**
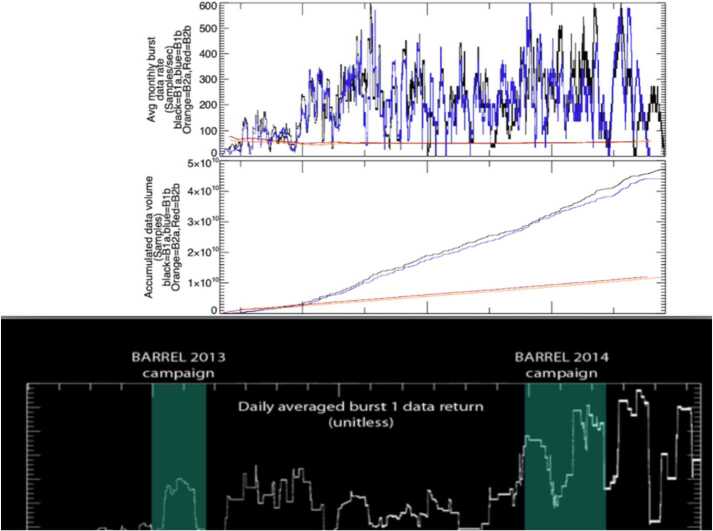
Burst 1 and 2 timelines. Panel **a** shows the average monthly data rate in samples/sec for both burst 1 (blue) and burst 2 (red), and, for comparison, the inverted DST index (black). Panel **b** shows the accumulated data volume. The last panel is a zoomed-in view of the average monthly rate showing the significant increase in data volume following the adoption of the sprint methodology and the burst 1 visual memory management software

At lower collection speeds ($\sim512\text{--}2048~\text{samples/sec}$) the operation of the burst 1 memory was typically straightforward. Days of continuous data could be collected, and with playback rates of up to 1 hr per day the 30 GB burst memory was seldom at risk of being completely filled. In contrast, at the highest sample rate of 16,384 samples/sec a single hour of collection took up a sizable chunk of the memory and about four days to play back (Table 7, column c). Overcommitting to 16,384 samples/sec collection could quickly gridlock the memory, significantly limiting options for the collection of further, possibly more interesting data. Early in the mission gridlocking was avoided by using a highly conservative approach to data collection, reducing the telemetry of interesting data. This was made particularly evident during the first collaborative campaign with the BARREL balloon mission in 2013. This experience showed that during times of intensive collection efforts managing the EFW burst 1 memory was very labor intensive, was associated with a high risk of mistakes, and limited the return of scientifically interesting data. EFW addressed these issues when collecting at high collection speeds by adopting a sprint burst collection methodology and a visual based memory management software package. With these enhancements, along with experience gained along the way, the daily averaged burst data return more than tripled. This is shown in the timeline plots in Fig. 41 as the sudden increase in averaged daily (panel c) and monthly (panel a) telemetry and increase in the slope in the total accumulated burst data volume (panel b). The sprint burst collection methodology and visual software are explained in the following subsections.

##### Sprint Burst Collection Methodology

The sprint methodology was designed to significantly increase the amount of burst data collected while avoiding memory gridlock by identifying data worth telemetering based on real-time space weather indices and predictive models rather than on-orbit survey data. The survey data were available only after a 2–3-day delay, which was longer than the time it took to fill the burst memory when collecting at 16,384 samples/sec. The sprint methodology involved the following:

1. Continuous collection of data at 16,384 samples/sec (typically within $\pm2.5$ hours of each apogee), and occasional lower rate collection near perigee.
2. Use of real-time space weather indices or predictive models to predict what parts of stored memory likely contained interesting wave data.
3. Protecting these memory locations against future overwriting.
4. Thoughtful selection of which data to telemeter in order to prevent a large backlog of playback requests which would limit future collection.

This real-time decision-making capability meant that long durations (typically $\sim5~\text{hrs}$) of 16,384 samples/sec burst data could be collected at every apogee, and times of potentially interesting data could be flagged and protected before being at risk of being overwritten by a future collection. This approach had the advantage of significantly increasing collection capability, but with the tradeoff of relying on predictive models (rather than actual survey data) to flag interesting times.

The sprint approach was used successfully for the majority of the Van Allen Probe mission and was a significant factor in increasing the volume of telemetered data as indicated in Fig. 41.

##### Visual Software

**Fig. 42:**
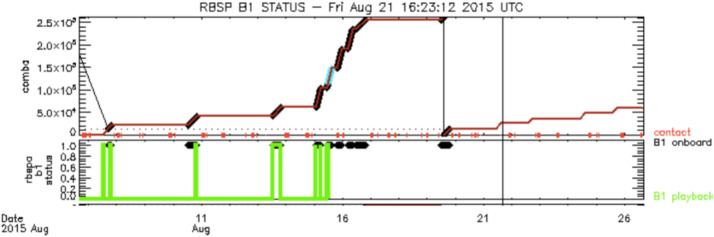
Example of the timeline output of the EFW burst memory manager software for RBSPa. (**a**) Red lines show the future prediction of the memory pointer location, thick black lines show currently recorded data, and thick blue lines show locations of potentially interesting data that have been designated as protected from overwrite. Spacecraft contacts are shown as the orange ticks, while the vertical line indicates the last time the code was run. (**b**) The thick black lines are a flattened version of those in panel (**a**), and the area under the green lines indicates data that has been telemetered

In order to reduce the required efforts for burst 1 operation the EFW team developed a visual burst memory control software package that significantly automated the workflow of managing the burst 1 memory. Figure 42 shows an example of the software’s visual output. The top panel indicates information related to the circular burst memory, with the y-axis representing the position in memory. The red curve is the historical trace of the predicted record pointer location as future collection was requested. The thick black lines show actual recorded data, while the thick blue lines show data that has been scheduled for future playback. This presents a clear visual indicator allowing the duty scientist to easily manage the burst 1 memory.

This software was typically run on a daily basis, giving the Duty Scientist the most up to date picture of the burst memory and allowing them to maximize the return of scientifically useful burst data.

The software was first used at the start of the second BARREL campaign in 2014. Its effect can clearly be seen by the distinct increase in daily averaged burst 1 data return seen in the last panel of Fig. 41. This early success set a trend for later collaborations, and this higher data return rate was largely maintained throughout the rest of the mission. Following this approach, EFW could go on to collect an unprecedented dataset of burst waveform data.

As previously discussed, the sprint approach was very labor intensive and stressful. In order to alleviate the required efforts and avoid Tohban burnout and errors the EFW team developed a visual burst memory control software package. This automated a majority of calculations needed to schedule collection and playback, and allowed recording and playback indicators to be predicted with high accuracy over days to weeks, significantly decreasing the Tohban workload. As the above plot shows, this sprint approach and software, implemented prior to the start of the 2014 BARREL campaign, led to significant increases in the rate of data return over time. This early success set a trend for later collaborations, and this higher data return rate was largely maintained throughout the rest of the mission. This huge flexibility and capability allowed for a wide range of dynamic campaigns to be undertaken depending on collection needs (rate, location, occurrence).

Following this approach, EFW could go on to collect an unprecedented dataset of burst waveform data during times of conjunctions, and would set a precedent that would be adopted by MMS. During the FIREBIRD and BARREL campaigns this included hundreds of hours of burst data (see Reeves et al. in prep).

### Radiation Belt Storm Probes Ion Composition Instrument (RBSPICE)

#### Data Production Planning Required Upgrades to Server and Data Systems

RBSPICE production system hardware was underspecified for the needs of production for a seven-year mission. The nominal RBSP mission was identified as a two-year mission but the fuel tank was “topped off” so that the mission could extend as long as maneuvering fuel was available. The RBSPICE SOC production hardware was purchased based upon the nominal mission and expected telemetry rates. After the third year of production it became clear to the SOC operations team that the hardware required upgrades as well as the operating systems to allow the overall daily production to occur within a reasonable time frame. As the necessity for reprocessing occurred during the mission due to revised calibrations or identified software defects, the production system hardware was taxed beyond its capabilities. This required purchase of additional hardware for reprocessing activities. Toward the end of the mission, system virtualization was used to handle the required hardware scaling issues. Virtualization was done using a Hyper-V system running on DELL server R720 hardware capable of running a total of ten Virtual Machines (VMs) – five per spacecraft. In the last year of extended mission II, the total reprocessing effort going from telemetry through Level 3 PAP took approximately 2.5 months. After this particular reprocessing effort was finished the DELL hardware was upgraded to a DELL R840 allowing for 20 VMs per spacecraft for full reprocessing efforts reducing the reprocessing effort total time to approximately 1/2–1 month.

#### Telemetry Volume Was Significantly Larger than Planned

RBSPICE instrument telemetry minimum bitrate to meet the science goals was specified at 1.565 Kbps and the nominal telemetry rate was 3.935 Kbps. During the course of the mission the Mission Operations Center (MOC) team became comfortable that the overall total volume from each spacecraft was not exceeding key thresholds and later in the mission the MOC team released reserve bandwidth for science telemetry. This increased the total RBSPICE bandwidth from $\sim1.5~\text{kbps}$ to $\sim3.9~\text{kbps}$. The impact of the transition from the nominal bandwidth to the end of mission bandwidth increased the overall data load on the SOC production systems requiring upgrading from a single stack of $10\times4~\text{TB}$ hard disk drives (HDD for 40 TB total) to 4 stacks of $10\times4~\text{TB}$ (160 TB) HDDs and a $32\times2~\text{TB}$ (64 TB) HDDs SAN system to provide for redundancy, backup, and offline disaster recovery. The final configuration added another $20\times4~\text{TB}$ HDDs (80 TB) and $24\times1.62~\text{TB}$ NVMe drives (NMD) (38.8 TB) for a tiered drive system providing for faster production coupled with higher throughput.

#### Programming of the Nominal RBSPICE Virtual Spin Period During Eclipse

The RBSPICE virtual spin system programmed into the instrument flight software had difficulty maintaining the true spacecraft spin during eclipses. The nominal spin period for each spacecraft programmed into the software was 12 sec. The RBSPICE flight software was designated as NASA Computer Software Configuration Item (CSCI) Class B software with the specification of “Non-Human Space-Rated Software Systems”. The Class B designation exists to prevent and/or significantly reduce the potential impact of the introduction of a software defect into operational flight software. It was determined to not change this software because the virtual spin period existed as part of the flight software and making the change was determined to expensive for the benefit.

The flight software spin virtualization worked exceptionally well under this configuration except for times in which the spacecraft would go into an eclipse for durations that exceeded several spin periods. The actual spin period of each spacecraft was approximately 10.9 seconds although this period varied throughout the mission. Since the spacecraft were configured as a sun pointing spacecraft, every three weeks each spacecraft required commanding to adjust orbital pointing to reduce the sun pointing angle and prevent instruments from having a direct UV exposure. Each commanded adjustment caused the spin period to reduce slightly and after a year or more of operations the MOC would need to command each spacecraft with spin-down operations in order to reduce the spin rate (increase the spin period). These spin-down operations occurred five times for SC A on Mission Days (MD) 257 (5/13/13), 624 (5/15/14), 1086 (8/20/15), 1651 (3/7/17), and 2373 (2/27/19-deorbit burn); and twice on SC B on MDs 1086 (8/20/15), and 2493 (6/27/19-deorbit burn).

**Fig. 43:**
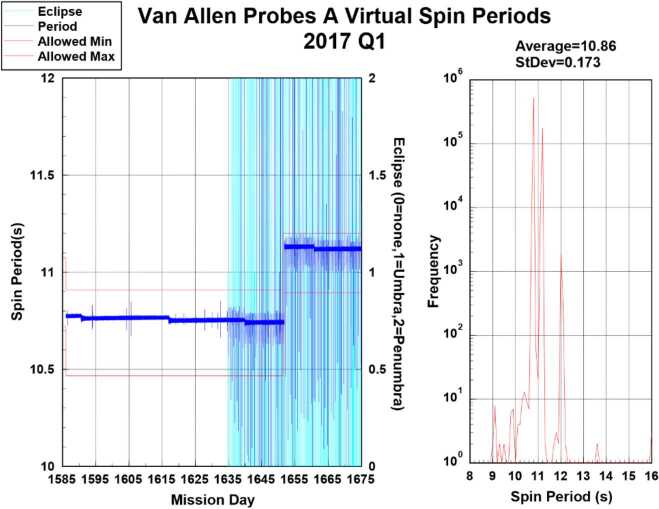
RBSPICE software defined virtual spin (dark blue) is shown in the left pane displaying the variation of the virtual spin before an eclipse period (white background) and during an eclipse period (cyan background) where the out of nominal virtual spin values increased dramatically during the eclipse period so that some data collected during this period has pointing that is no longer valid for lack of understanding of the pointing of the instrument during these times. The right pane presents the distribution of spin periods over the time frame of the left pane. This plot shows that the hard-coded 12 second spin period occurs fairly frequently but also that much lower and higher spin periods occur when the virtual spin software is either slowing down or catching up to the real spin period post-eclipse

During an eclipse the RBSPICE flight software would automatically switch from the nominal $\sim10.9~\text{sec}$ spin period to a 12 second spin period. If this spin-to-spin offset continued for more than a few spins then the spacecraft pointing information became unreliable as the software would indicate that the instrument was in a sector that was mismatched with respect to the actual instrument pointing. Figure 43 displays an example of the RBSPICE virtual spin period for the first quarter of 2017. The dark blue curve in the left panel plots record by record values of the virtual spin period of the flight software and the light blue colored shaded areas show periods where the spacecraft would include an eclipse within the orbit. The plot is done during a time when the spacecraft is commanded during MD 1651 to do a spin down maneuver increasing the spin period. The variation of the virtual spin is very small while the spacecraft is outside an eclipse period but has significant variation during eclipse periods. The right-side panel displays the distribution of spin periods showing that the majority are well within the nominal spacecraft spin range. That figure also shows a smaller number of times when the coded 12 sec period is utilized during an eclipse and when the spacecraft exits the umbra of the eclipse and reacquires the sun then the flight software does some dramatic changes in the virtual spin period to either slow down or catch up to the actual spacecraft spin. The number of spins required to resynch the virtual spin with the actual spin was less than two or three spins. During the times when the virtual spin was out of synch with the spacecraft spin, the accumulation records were tagged by the flight software with sector numbers shifted in phase compared to the actual spacecraft pointing. During these times the quality flags of the Level 3 and above RBSPICE data is tagged as bad.

## Appendix

**Table 8:** NASA Science Data Level Specifications

NASA	CODMAC	Description
Packet data	Raw – Level 1	Telemetry data stream as received at the ground station, with science and engineering data embedded.
Level 0	Edited – Level 2	Instrument science data (e.g., raw voltages, counts) at full resolution, time ordered, with duplicates and transmission errors removed.
Level 1A	Calibrated – Level 3	Level 0 data that have been located in space and may have been transformed (e.g., calibrated, rearranged) in a reversible manner and packaged with needed ancillary and auxiliary data (e.g., radiances with the calibration equations applied).
Level 1B	Resampled – Level 4	Irreversibly transformed (e.g., resampled, remapped, calibrated) values of the instrument measurements (e.g., radiances, magnetic field strength).
Level 1C	Derived – Level 5	Level 1A or 1B data that have been resampled and mapped onto uniform space-time grids. The data are calibrated (i.e., radiometrically corrected) and may have additional corrections applied (e.g., terrain correction).
Level 2	Derived – Level 5	Geophysical parameters, generally derived from Level 1 data, and located in space and time commensurate with instrument location, pointing, and sampling.
Level 3	Derived – Level 5	Geophysical parameters mapped onto uniform space-time grids.

## Footnotes

1 Tohban: (org.) Japanese (def.) operator; duty officer per RHESSI operations manual, sprg.ssl.berkeley.edu/~tohban/wiki/index.php/Tohban Earliest reference for Tohban for Space Science is from “Tohban Manual” for Yohkoh Toban duties at Institute of Space and Astronautical Science part of Japan Aerospace Exploration Agency (JAXA), 16 Oct. 1995 and is since a title used in multiple space missions include EFW referring to daily or weekly person in charge of operations and responsible for spacecraft/instrument health and safety or the “Duty Officer”.
